# Effect of Levothyroxine Therapy on the Development of Depressive Symptoms in Older Adults With Subclinical Hypothyroidism: An Ancillary Study of a Randomized Clinical Trial

**DOI:** 10.1001/jamanetworkopen.2020.36645

**Published:** 2021-02-10

**Authors:** Lea Wildisen, Martin Feller, Cinzia Del Giovane, Elisavet Moutzouri, Robert S. Du Puy, Simon P. Mooijaart, Tinh-Hai Collet, Rosalinde K. E. Poortvliet, Patricia Kearney, Terence J. Quinn, Stefan Klöppel, Douglas C. Bauer, Robin P. Peeters, Rudi Westendorp, Drahomir Aujesky, Jacobijn Gussekloo, Nicolas Rodondi

**Affiliations:** 1 Institute of Primary Health Care (BIHAM), University of Bern, Bern, Switzerland; 2 Graduate School for Health Sciences, University of Bern, Bern, Switzerland; 3 Department of General Internal Medicine, Inselspital, Bern University Hospital, University of Bern, Bern, Switzerland; 4 Department of Public Health and Primary Care, Leiden University Medical Center, Leiden, the Netherlands; 5 Department of Gerontology and Geriatrics, Leiden University Medical Center, Leiden, the Netherlands; 6 Service of Endocrinology, Diabetology, Nutrition, and Therapeutic Education, Geneva University Hospitals, Geneva, Switzerland; 7 School of Public Health, University College Cork, Cork, Ireland; 8 Institute of Cardiovascular Medicine, University of Glasgow, Glasgow, Scotland; 9 University Hospital of Old Age Psychiatry, University of Bern, Bern, Switzerland; 10 Departments of Medicine and Epidemiology and Biostatistics, University of California, San Francisco, San Francisco, California; 11 Department of Medicine, Erasmus Medical Center, Rotterdam, the Netherland; 12 Department of Public Health and Center for Healthy Aging, University of Copenhagen, Copenhagen, Denmark

## Abstract

**Importance:**

Previous trials on the effect of levothyroxine on depressive symptom scores in patients with subclinical hypothyroidism were limited by small sample sizes (N = 57 to 94) and potential biases.

**Objective:**

To assess the effect of levothyroxine on the development of depressive symptoms in older adults with subclinical hypothyroidism in the largest trial on this subject and to update a previous meta-analysis including the results from this study.

**Design, Setting, and Participants:**

This predefined ancillary study analyzed data from participants in the Thyroid Hormone Replacement for Untreated Older Adults with Subclinical Hypothyroidism (TRUST) trial, a double-blind, randomized, placebo-controlled, parallel-group clinical trial conducted from April 2013 to October 31, 2016. The TRUST trial included adults aged 65 years or older diagnosed with subclinical hypothyroidism, defined as the presence of persistently elevated thyroid-stimulating hormone (TSH) levels (4.6-19.9 mIU/L) with free thyroxine (T4) within the reference range. Participants were identified from clinical and general practitioner laboratory databases and recruited from the community in Switzerland, the Netherlands, Ireland, and the UK. This ancillary study included a subgroup of 472 participants from the Netherlands and Switzerland; after exclusions, a total of 427 participants (211 randomized to levothyroxine and 216 to placebo) were analyzed. This analysis was conducted from December 1, 2019, to September 1, 2020.

**Interventions:**

Randomization to either levothyroxine or placebo.

**Main Outcomes and Measures:**

Depressive symptom scores after 12 months measured with the Geriatric Depression Scale (GDS-15), with higher scores indicating more depressive symptoms (minimal clinically important difference = 2).

**Results:**

A total of 427 participants with subclinical hypothyroidism (mean [SD] age, 74.52 [6.29] years; 239 women [56%]) were included in this analysis. The mean (SD) TSH level was 6.57 (2.22) mIU/L at baseline and decreased after 12 months to 3.83 (2.29) mIU/L in the levothyroxine group; in the placebo group, it decreased from 6.55 (2.04) mIU/L to 5.91 (2.66) mIU/L. At baseline, the mean (SD) GDS-15 score was 1.26 (1.85) in the levothyroxine group and 0.96 (1.58) in the placebo group. The mean (SD) GDS-15 score at 12 months was 1.39 (2.13) in the levothyroxine and 1.07 (1.67) in the placebo group with an adjusted between-group difference of 0.15 for levothyroxine vs placebo (95% CI, −0.15 to 0.46; *P* = .33). In a subgroup analysis including participants with a GDS-15 of at least 2, the adjusted between-group difference was 0.61 (95% CI, −0.32 to 1.53; *P* = .20). Results did not differ according to age, sex, or TSH levels. A previous meta-analysis (N = 278) on the association of levothyroxine with depressive symptoms was updated to include these findings, resulting in an overall standardized mean difference of 0.09 (95% CI, −0.05 to 0.22).

**Conclusions and Relevance:**

This ancillary study of a randomized clinical trial found that depressive symptoms did not differ after levothyroxine therapy compared with placebo after 12 months; thus, these results do not provide evidence in favor of levothyroxine therapy in older persons with subclinical hypothyroidism to reduce the risk of developing depressive symptoms.

**Trial Registration:**

ClinicalTrials.gov Identifier: NCT01853579

## Key Points

**Question:**

Does levothyroxine treatment have an effect on the development of depressive symptoms in older adults with subclinical hypothyroidism?

**Findings:**

In this ancillary study of a randomized, placebo-controlled clinical trial of 427 participants with subclinical hypothyroidism, there was no statistically significant difference in the adjusted between-group difference in the mean Geriatric Depression Score at 12 months between the levothyroxine and placebo groups.

**Meaning:**

These results do not provide evidence in favor of levothyroxine therapy in older persons with subclinical hypothyroidism to reduce the risk of developing depressive symptoms.

## Teaser

This ancillary study of the Thyroid Hormone Replacement for Untreated Older Adults with Subclinical Hypothyroidism (TRUST) randomized clinical trial assesses whether levothyroxine treatment vs placebo has an effect on the development of depressive symptoms in older adults with subclinical hypothyroidism.

## Introduction

Subclinical hypothyroidism is defined as elevated thyroid-stimulating hormone (TSH) levels in combination with free thyroxine (T4) levels within the reference range.^1^ Subclinical hypothyroidism is a frequent condition in the general population, in particular among women and older adults,^2^ with a prevalence up to 10% to 15%.^3,4^ Increasing evidence suggests that patients with subclinical hypothyroidism should not routinely be treated with levothyroxine.^5,6,7^ Current guidelines recommend levothyroxine therapy for adults with TSH levels greater than 10 mIU/L and for people with lower TSH values who are young, symptomatic, or have specific indications for prescribing.^8,9^ Levothyroxine has become the most prescribed drug in the US in 2014 and the second most prescribed drug in the UK in 2019.^10,11^ More frequent testing or lower TSH thresholds could be potential reasons for the increase in levothyroxine prescription.^1^

Among other indicators, depressive symptoms are a common reason for starting levothyroxine therapy in patients with subclinical hypothyroidism.^12^ A recent meta-analysis of 4 trials on the effect of levothyroxine therapy in adult patients with subclinical hypothyroidism found no benefit for depressive symptoms,^7^ but data were limited by small sample sizes (N = 57-94) and potential biases, such as risk of bias assessment and publication bias.^7^

The Thyroid Hormone Replacement for Untreated Older Adults with Subclinical Hypothyroidism (TRUST) trial was a randomized, multicenter, double-blind placebo-controlled trial on the benefits of levothyroxine for patients 65 years or older with subclinical hypothyroidism.^6,13^ Our study aimed, first, to assess the possible effect of levothyroxine therapy on depressive symptom scores in older adults with subclinical hypothyroidism from the TRUST trial, and second, to update the previous meta-analysis^7^ on the effect of levothyroxine therapy on depressive symptoms scores including the results from the TRUST trial.^6^

## Methods

This ancillary study is nested in a large, international study on levothyroxine therapy in older adults with subclinical hypothyroidism (the TRUST trial), which was conducted from April 2013 to October 31, 2016.^6,13^ This ancillary study on depressive symptoms was predefined and registered in May 2013 separately from the main TRUST trial.^14^ In participants from 2 countries of the TRUST trial, Switzerland and the Netherlands, depressive symptoms were measured at baseline and at 12-month follow-up using the 15-item Geriatric Depression Scale (GDS-15). In a secondary analysis on incidence of mild depression, participants from the TRUST site in Ireland were included. In Ireland, depressive symptoms were measured by using the Center for Epidemiologic Studies Depression 20-item scale (CESD-20). The protocol of the TRUST trial was accepted by the relevant ethics committees and was published previously.^13^ Participants provided written consent to participate. The analysis plan for this ancillary study was accepted by the TRUST publication committee. This study followed the Consolidated Standards of Reporting Trials (CONSORT) reporting guideline for clinical trials.^15^

### The TRUST Trial

The main goal of the TRUST trial was to determine whether levothyroxine provides clinical benefits in older persons with subclinical hypothyroidism.^6^ The TRUST trial protocol is available in Supplement 1. In the TRUST trial, patients underwent randomization in a 1:1 ratio, with stratification according to country, sex, and starting dose, with the use of randomly permuted blocks.^6^ The study medication consisted of levothyroxine sodium tablets and matching placebo tablets taken orally once daily. The active intervention started with levothyroxine at a dose of 50 μg daily (or 25 μg in patients with a body weight of less than 50 kg or with known coronary heart disease, ie, previous myocardial infarction or symptoms of angina pectoris), and the placebo group started with a matching placebo for 6 to 8 weeks. The dose of levothyroxine was adjusted in 25-μg increments based on TSH levels measured 6 to 8 weeks after starting the intervention, 6 to 8 weeks after each dose adjustment, and at a 12-month follow-up in order to reach a TSH level within the reference range (0.4-4.6 mIU/L) in the levothyroxine group. An identical schedule for adjusting the dosage of placebo with mock titration was used to achieve an approximately equal frequency of dosage adjustments between the groups to maintain blinding. The participants, investigators, and treating physicians were unaware of the results of TSH measurements throughout the course of the trial and remained blinded for treatment allocation.^6^

### Participants

Potential participants aged 65 or older were identified from clinical and general practitioner laboratory databases and recruited from the community in Switzerland, the Netherlands and Ireland. Subclinical hypothyroidism was defined as the presence of persistently elevated TSH levels (4.6-19.9 mIU/L) at a minimum of 2 occasions at least 3 months apart with free T4 within the reference range. Participants were followed up for a minimum of 12 months. Exclusion criteria included the use of levothyroxine, antithyroid medication, amiodarone, or lithium; recent hospitalization for major illness; recent acute coronary syndrome, acute myocarditis, or pancreatitis; and terminal illness.

### Depressive Symptoms

Depressive symptoms were measured using the 15-item Geriatric Depression Scale (GDS-15) at baseline and 12-month follow-up. The GDS-15 is a well-validated test for depression screening in older age, with validity to measure longitudinal changes.^16,17,18^ The GDS-15 score ranges from 0 to 15, with higher scores indicating higher likelihood of depression. Score values from 0 to 2 indicate no depressive symptoms; 3-5, mild depressive symptoms; and 6 or greater, severe depressive symptoms.^19^ The minimal clinically important between-group difference on the GDS-15 is 2 points.^18^ In this study, we used the cutoff of 3, suggesting mild depression.^18^ One of the advantages of the GDS-15 is that it relies less on the somatic symptoms of depression and has been proven valid for use in patients with chronic physical diseases, as it is often the case in older adults.^16^ Visits with GDS-15 took place from April 2013 to May 2015 for the baseline assessment and from April 2014 to October 31, 2016, for the 12-month follow-up.

We defined the primary outcome as GDS-15 score at 12 months, adjusted for scores at baseline, age, sex, country, and starting dose of levothyroxine. As a secondary analysis, we dichotomized the depression scale using established cutoff values for depressive symptoms; for the GDS-15 score, a cutoff score greater than 3 suggests mild depression.^16^ For this analysis, we additionally included data from the TRUST site in Ireland, where depressive symptoms were measured using the CESD-20. This is a self-rating scale that measures depressive symptoms during the past week. The scale ranges from 0 to 60, with higher scores indicating more depressive symptoms. CESD-20 values from 16 to 20 indicate mild depressive symptoms, values from 20 to 25 indicate moderate symptoms, and values of 25 and greater indicate severe depressive symptoms.^20^ In this study, we used the cutoff of 20 to define the presence of depression.^21^ The CESD-20 has been shown to be a valid and reliable instrument to measure depressive symptoms and has been widely used in older populations.^22^

### Statistical Analysis

**Figure 1.**
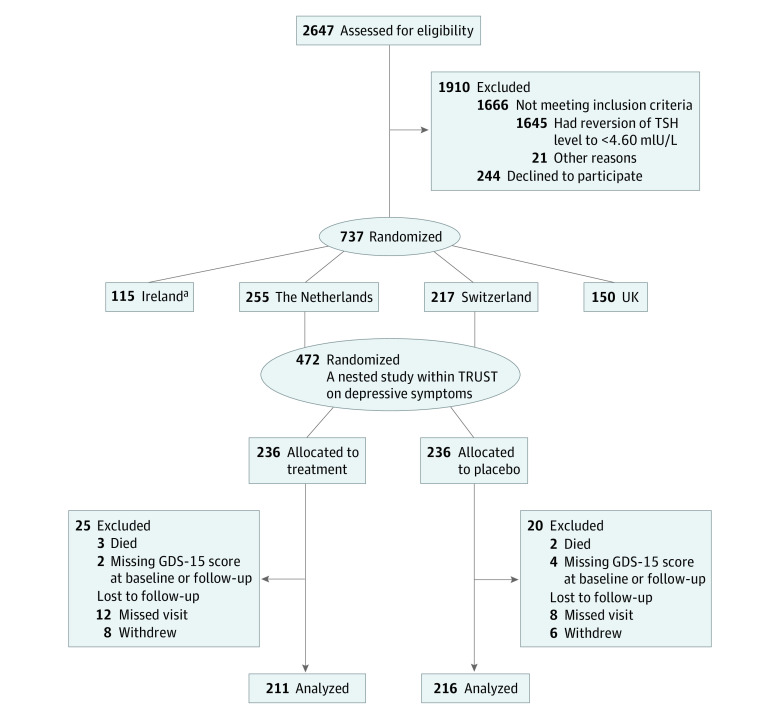
Flowchart of Study Participants In the Thyroid Hormone Replacement for Untreated Older Adults with Subclinical Hypothyroidism (TRUST) study sites in the Netherlands and Switzerland, depressive symptoms were measured using the Geriatric Depression Scale (GDS-15). TSH indicates thyroid stimulating hormone. ^a^In 87 participants from Ireland, depressive symptoms were measured using the Center for Epidemiologic Studies Depression 20-item scale (CESD-20); those participants were included in a secondary analysis on incidence of mild depression. In the UK, no depressive symptoms were measured.

Analyses were conducted from December 1, 2019, to September 1, 2020. For the primary outcome analysis, we performed a modified intention-to-treat analysis using those participants having depressive symptoms outcomes (Figure 1). Using the available data set, we performed a power calculation with 427 participants with a standard deviation of 2 and a mean GDS-15 score in control of 1, which resulted in 100% power for detecting a mean difference of 2.0 points at a 1-sided α-level of .05. We used a multivariable linear regression analysis to assess the mean difference and relative 95% CI in GDS-15 scores at the 12-month follow-up between the levothyroxine and placebo groups. We adjusted the results for GDS-15 scores at baseline, age, sex, country, and starting dose of levothyroxine.

In a secondary analysis, we first excluded participants with mild depression at baseline (indicated by GDS-15 scores >3 or CESD-20 scores >20)^16,23^ and analyzed those who developed mild depression during follow-up in both the levothyroxine and placebo groups. Second, we included only participants with mild depression at baseline and analyzed those who recovered from mild depression. We conducted both secondary analyses (1) only in participants from Switzerland and the Netherlands and (2) including participants from Ireland (n = 87). We used a multivariable logistic regression model to assess odds ratios (ORs) and 95% CIs adjusted for depressive symptoms scores at baseline, age, sex, country, and starting dose of levothyroxine.

In subgroup analyses, we assessed the difference in GDS-15 scores at 12 months separately for men and women, for participants above and below 75 years of age, for different TSH levels (4.5 to <7.0, 7.0 to <10.0, and 10.0-20 mIU/L), and for participants with GDS-15 scores above and below 2, as the minimal clinically relevant difference on the GDS-15 scale is 2 (first clinically relevant difference from 0).^18^

We excluded participants without depressive symptom measurements (Figure 1). We used ipsative imputation for participants with partially incomplete GDS-15 questionnaires,^24^ which means we divided the number of positive answered questions by the number of not-missing questions and multiplied this by 15 (amount of questions of the GDS-15 scale ÷ maximal score). This calculation is a valid imputation method to impute data from the 15-item GDS scale if the percentage of its missing items is 20% or less.^24^ From 427 participants (211 in treatment and 216 in the placebo group) with available data on depressive symptoms, 4 participants had an incomplete GDS-15 questionnaire at baseline, and 8 had an incomplete GDS-15 questionnaire at 12-month follow-up (only 1 participant with more than 20% missing answers was included).

We conducted several sensitivity analyses to test the robustness of the results. In the main analysis, we used a parametric model, because it has several advantages compared to nonparametric models and because parametric models have been shown to perform statistically well if the study size is big enough (n > 25).^25^ To verify, we also applied a nonparametric model (Kernel regression) in a sensitivity analysis.^26^ In another sensitivity analysis, we excluded participants with antidepressant treatment (Anatomical Therapeutic Chemical Classification System: N06A, n = 26). In a sensitivity analysis, we imputed missing GDS-15 score at baseline or follow-up (n = 45). We imputed data under the assumption of missing at random using multivariate regression imputation based on an individual’s complete responses on the variables of sex, age, country, and starting dose of levothyroxine. We additionally simulated a scenario assuming all missing GDS-15 scores at 12 months to be 75% higher (imputed values multiplied by 1.75) and a worst-case scenario assuming all participants with missing GDS-15 score in the placebo group to have a maximal score of 15 points at 12 months, whereas we assumed that participants with missing scores in the treatment group had a score of 0 at 12 months.

#### Update of Meta-analysis

As this study is, to our knowledge, the biggest trial to investigate the effect of levothyroxine on depressive symptoms in patients with subclinical hypothyroidism, we also updated a recent meta-analysis on 278 participants with the results from this ancillary study on the TRUST trial on 427 participants.^7^ Because studies used different scores to assess depressive symptoms, we pooled standardized mean differences (SMDs) using a random effects model. A negative SMD indicates benefit of levothyroxine therapy, with −0.2, −0.5, and −0.8 corresponding to small, moderate, and large effects, respectively.^27^ Heterogeneity was assessed with *I*^2^ statistics (might not be important: 0%-40%; moderate: 30%-60%; substantial: 50%-90%; considerable: >75%).^27^ Two reviewers (L.W. and M.F.) independently evaluated the quality of evidence using the Grading of Recommendations, Assessment, Development and Evaluation (GRADE) tool.^28^ The certainty of evidence reflects the extent to which the confidence in the estimate of the effect is adequate to support a recommendation regarding levothyroxine therapy in participants with subclinical hypothyroidism and depressive symptoms.

In a sensitivity analysis, we excluded studies that were only conducted in elderly participants from the meta-analysis.

All analyses were conducted with STATA version 16 (StataCorp LLC) for Windows.

## Results

A total of 427 participants were included in the main analysis (mean age [SD] age, 74.52 (6.29) years; 239 women [56.0%]) (Table 1). The mean (SD) TSH level was 6.57 (2.22) mIU/L at baseline and decreased after 12 months to 3.83 (2.29) mIU/L in the levothyroxine group; in the placebo group, it decreased from 6.55 (2.04) mIU/L to 5.91 (2.66) mIU/L. At baseline, the mean (SD) GDS-15 score was 1.26 (1.85) in the levothyroxine group and 0.96 (1.58) in the placebo group. The mean (SD) GDS-15 score at 12 months was 1.39 (2.13) for levothyroxine group and 1.07 (1.67) for the placebo group with an adjusted between-group difference of 0.15 (95% CI, −0.15 to 0.46; *P* = .33; minimal clinically relevant difference = 2.0)^18^ (Table 2).

**Table 1.** Characteristics of Participants at Baseline

Characteristic	No. (%)	
	Levothyroxine (n = 211)	Placebo (n = 216)
Age, mean (range), y	73.99 (65.37-91.17)	75.04 (65.12-93.40)
Women	118 (56)	121 (56)
Previous medical conditions and clinical descriptors		
Atrial fibrillation	28 (13)	23 (11)
Hypertension	102 (48)	98 (46)
Diabetes	34 (16)	26 (12)
Osteoporosis	26 (13)	31 (15)
Current smoking	18 (9)	19 (9)
Dementia	0	0
Excess alcohol consumption^a^	2 (1)	3 (1)
Antidepressants medication	16 (8)	10 (5)
Mini-Mental State Examination score, mean (SD)	28.52 (1.39)	28.68 (1.42)
Weight		
Mean (range), kg	77.58 (46-150)	76.76 (44-121)
<50 kg	4 (2)	3 (1)
BMI, mean (SD)	27.88 (5.54)	27.60 (4.37)
TSH, mean (SD) [range], mIU/L	6.57 (2.22) [4.60-17.58]	6.55 (2.04) [4.60-17.60]
Free T4, mean (SD) [range], pmol/L	13.69 (1.97) [10.00-20.60]	13.61 (1.86) [9.00-21.90]
GDS-15 score, mean (SD) [range]	1.26 (1.85) [0-9]	0.96 (1.58) [0-12]

Abbreviations: BMI, body mass index (calculated as weight in kilograms divided by height in meters squared); GDS-15, 15-item Geriatric Depression Scale Questionnaire (range, 0-15; higher scores indicate more severe depressive symptoms; minimal clinically important difference, 2 points); TSH, thyroid stimulating hormone; T4, thyroxine.

^a^ More than 35 units per week for women and more than 50 units per week for men.

**Table 2.** Difference in GDS-15 Score at 12 Months Between Levothyroxine and Placebo Groups

Mean (SD) GDS-15 score				Unadjusted mean difference at 12 mo (95% CI)^a^	*P* value	Mean difference at 12 mo, djusted for age and sex (95% CI)^a^	*P* value	Fully adjusted mean difference at 12 mo (95% CI)^a^^,^^b^	*P* value
Baseline		12 mo							
Levothyroxine (n = 211)	Placebo (n = 216)	Levothyroxine (n = 211)	Placebo (n = 216)						
1.26 (1.85)	0.96 (1.58)	1.39 (2.13)	1.07 (1.67)	0.32 (−0.05 to 0.68)	.09	0.35 (−0.02 to 0.71)	.06	0.15 (−0.15 to 0.46)	.33

Abbreviations: GDS-15, 15-item Geriatric Depression Scale Questionnaire (range, 0-15; higher scores indicate more severe depressive symptoms; minimal clinically important difference, 2 points).

^a^ Positive results indicate benefit of placebo.

^b^ Adjusted for age, sex, GDS-15 score at baseline, levothyroxine dose at baseline, and country.

Of the 472 participants from the Netherlands and from Switzerland, 236 were allocated to levothyroxine and 236 to the placebo group (Figure 1). Forty-five participants were excluded from the analysis (5 participants died before the 12-month visit, 6 had a missing GDS-15 score at baseline or the 12-month visit, 20 missed the 12-month visit, and 14 withdrew before the 12-month visit). The proportion of excluded participants was similar in the 2 groups (25 participants [11%] in the treatment group and 20 [8%] in the placebo group). Participants who were lost to follow-up (n = 34) had a higher mean (SD) GDS-15 score (1.80 [2.42]) at baseline compared with those with a completed follow-up (1.11 [1.72]). Finally, 427 participants were included in the main analysis of this ancillary study, with 211 receiving levothyroxine and 216 receiving the placebo. The baseline characteristics of the 45 excluded participants were similar to those who were included (eTable 1 in Supplement 2).

**Figure 2.**
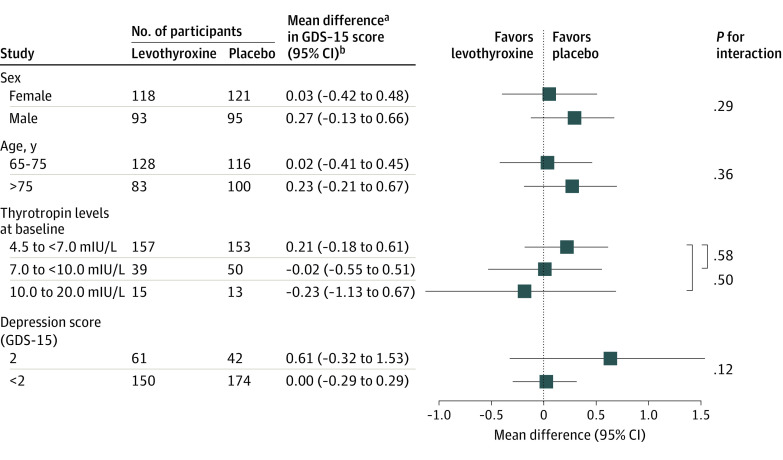
Subgroup Analyses: Difference in GDS-15 Score at 12 Months Between Levothyroxine and Placebo Groups GDS-15 indicates Geriatric Depression Scale 15-item questionnaire (range, 0-15; higher scores indicate more severe depressive symptoms; minimal clinically important difference, 2 points). ^a^Positive results indicate benefit of placebo. ^b^Adjusted for age, sex, GDS-15 score at baseline, levothyroxine dose at baseline, and country.

There was no clinically relevant difference in depressive symptoms at 12 months in subgroup analyses by age, sex, and TSH levels (Figure 2), nor in participants with GDS-15 scores of 2 or higher at baseline (adjusted between-group difference, 0.61; 95% CI, −0.32 to 1.53; *P* = .20).

In a secondary analysis, we calculated the incidence of mild depression. At baseline, 188 patients in the levothyroxine group and 206 in the placebo group had no mild depression . During follow-up, 10 participants from the levothyroxine group (5.3%) and 12 participants from the placebo group (5.8%) developed mild depression (GDS-15 score >3). The OR for mild depression comparing the levothyroxine group vs the placebo group was 0.87 (95% CI, 0.36-2.13). We then repeated the analysis after adding 87 participants from Ireland with available data on CESD-20 scores, for an OR of 1.06 (95% CI, 0.45-2.49; values >1 indicate benefit of levothyroxine therapy) (eTable 2 in Supplement 2). When we only included participants with mild depression at baseline, the OR for recovery of mild depression was 0.20 (95% CI, 0.02-1.93; values <1 indicate benefit of placebo) (eTable 3 in Supplement 2).

Results stayed robust in a sensitivity analysis excluding participants with antidepressant medication at baseline (adjusted between-group difference, 0.14; 95% CI, −0.17 to 0.46) or using the Kernel-regression model (adjusted between-group difference, 0.12; 95% CI, −0.38 to 0.41). The results stayed robust when we imputed missing GDS-15 scores at baseline and follow-up and when we simulated missing values assuming the missing values to be 75% higher than nonmissing values. When we simulated a worst-case scenario for missing values at 12 months, the difference between the groups was below minimal clinically important difference (−1.17; 95% CI, −1.74 to −0.61).

**Figure 3.**
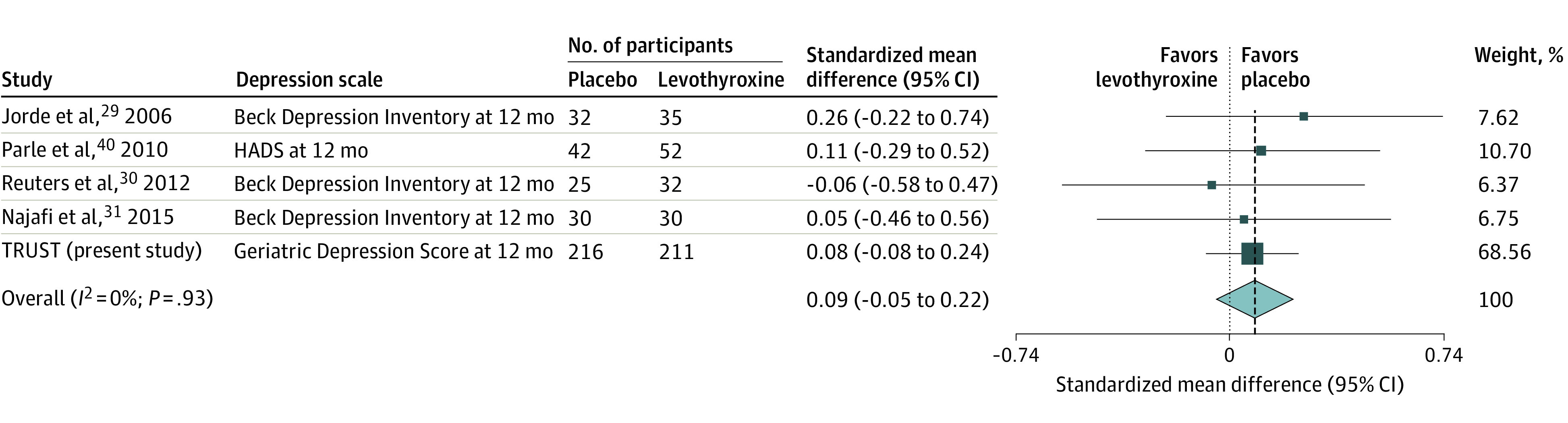
Update of Meta-analysis: Standardized Mean Differences Between Levothyroxine and Placebo Groups in Depressive Symptoms Weights are derived from a random-effects meta-analysis of standardized mean differences. Standardized mean differences of −0.2, −0.5, and −0.8 correspond to small, moderate, and large clinical positive effects of levothyroxine, respectively. The size of the boxes indicates the study weight in the meta-analysis, the whiskers represent 95% CIs, the diamond shows the result of the meta-analysis, and the vertical dashed line represents the point estimate of the pooled standardized mean difference. HADS indicates Hospital Anxiety and Depression Scale; TRUST, Thyroid Hormone Replacement for Untreated Older Adults With Subclinical Hypothyroidism.

To pool the results from this ancillary study on depressive symptoms within the TRUST trial to the previous meta-analysis,^7^ we transformed the adjusted mean difference in depressive symptoms between the 2 groups of 0.15 (95% CI, −0.15 to 0.46) to an adjusted standardized mean difference (SMD) of 0.08 (95% CI, −0.08 to 0.24). When we pooled the adjusted SMD with the previous meta-analysis,^7^ we found an overall SMD of 0.09 (95% CI, −0.05 to 0.22) (Figure 3). There was no statistical heterogeneity (*I*^2^ = 0.0%). In a sensitivity analysis including only 3 studies that were not only conducted in elderly participants, we found an overall SMD of 0.09 (95% CI, −0.20 to 0.38).^29,30,31^ This ancillary study on depression within a randomized controlled trial had a weight of 67.6% to the overall effect. The quality of evidence assessed with the GRADE tool was high, indicating that further research is very unlikely to change our confidence in the estimate of effect (eTable 4 in Supplement 2).

## Discussion

In this ancillary study within a randomized controlled trial among adults aged 65 years or older with subclinical hypothyroidism, levothyroxine therapy for 12 months had no clinical effect on depressive symptom scores. Nor was there a difference in the incidence of mild depression in the levothyroxine group compared with the placebo group after 12 months. The results stayed robust in several subgroup analyses. In this study, we additionally found that participants with slightly increased depression scores at baseline (GDS-15 ≥ 2) did not improve under levothyroxine therapy compared with placebo.

Observational studies on the association between subclinical hypothyroidism and depressive symptoms have yielded conflicting results. Several studies showed that participants with subclinical hypothyroidism had more severe depressive symptoms, but other studies reported no differences.^32,33,34,35,36,37,38^ However, our recently conducted individual participant data (IPD) analysis, including younger and older participants, revealed no prospective association between subclinical hypothyroidism and depressive symptoms.^39^ Previous clinical trials were rather small and could not find an effect of levothyroxine therapy on depressive symptoms.^29,30,31,40^

### Strengths and Limitations

This study has several strengths. First, the large sample size makes it the largest trial, to our knowledge, on depressive symptoms in patients with subclinical hypothyroidism. Second, we updated the previous meta-analysis to include this study. The effect estimate’s confidence interval excluded a possible effect of levothyroxine on the development of depressive symptoms in participants with subclinical hypothyroidism. Third, the quality of evidence assessed with the GRADE scale was high, which indicates that further research is very unlikely to change our confidence in the estimate of effect.^28^

This study has some limitations. First, GDS-15 scores were low in our study population at baseline as reflected in the general population. Therefore, we cannot exclude that patients with subclinical hypothyroidism and moderate or severe depressive symptoms might benefit from levothyroxine therapy. Second, participants lost to follow-up had a higher mean GDS-15 score at baseline compared to those with a completed follow-up, which suggests that participants with higher scores were more likely lost to follow-up. In a sensitivity analysis in which we imputed missing scores, assuming participants with missing scores to have 75% higher scores, results stayed robust. Third, as we only included participants aged 65 years or older, our results are not generalizable to younger patients with subclinical hypothyroidism. Fourth, this study on depressive symptoms was an ancillary study of the TRUST trial; therefore, we were not able to include all participants from the original study, as depressive symptoms were not measured at all sites of the TRUST trial. Fifth, depressive symptoms were self-reported.

## Conclusion

Current guidelines on subclinical hypothyroidism recommend taking into account age, TSH levels, and the presence of related symptoms when deciding whether to treat a patient with subclinical hypothyroidism with levothyroxine.^5,9,41^ This ancillary study of a randomized clinical trial found no effect of levothyroxine therapy on the development of depressive symptoms in older patients, even among those with a TSH level higher than 10 mIU/L, although this group was relatively small. Findings from this study do reinforce the current recommendation that levothyroxine therapy should not be prescribed to reduce the risk of depressive symptoms in adults with subclinical hypothyroidism, with high quality of evidence.^5^ However, uncertainty remains on the effect of the treatment among severely depressed patients, as this trial was only conducted in patients with rather low depressive symptom scores at baseline.

## Notes

Supplement 1. **Trial Protocol**

Supplement 2. **eTable 1.** Comparison of Baseline Characteristics Between Participants With Available Follow Up and Participants With Missing Follow Up **eTable 2.** Secondary Analyses: Incidence of Mild Depression **eTable 3.** Secondary Analyses: Recovery from Mild Depression **eTable 4.** Quality of Evidence (GRADE) on Effect on Levothyroxine Therapy in Non-pregnant Adults With Subclinical Hypothyroidism on Depressive Symptoms

Supplement 3. **Data Sharing Statement**

## References

[1] Biondi B, Cappola AR, Cooper DS Subclinical hypothyroidism: a review. JAMA. 2019;322(2):153-160. doi:10.1001/jama.2019.9052 31287527

[2] Canaris GJ, Manowitz NR, Mayor G, Ridgway EC The Colorado thyroid disease prevalence study. Arch Intern Med. 2000;160(4):526-534. doi:10.1001/archinte.160.4.526 10695693

[3] Cooper DS, Biondi B Subclinical thyroid disease. Lancet. 2012;379(9821):1142-1154. doi:10.1016/S0140-6736(11)60276-6 22273398

[4] Biondi B, Cooper DS The clinical significance of subclinical thyroid dysfunction. Endocr Rev. 2008;29(1):76-131. doi:10.1210/er.2006-0043 17991805

[5] Bekkering GE, Agoritsas T, Lytvyn L, Thyroid hormones treatment for subclinical hypothyroidism: a clinical practice guideline. BMJ. 2019;365:l2006. doi:10.1136/bmj.l2006 31088853

[6] Stott DJ, Rodondi N, Kearney PM, ; TRUST Study Group Thyroid hormone therapy for older adults with subclinical hypothyroidism. N Engl J Med. 2017;376(26):2534-2544. doi:10.1056/NEJMoa1603825 28402245

[7] Feller M, Snel M, Moutzouri E, Association of thyroid hormone therapy with quality of life and thyroid-related symptoms in patients with subclinical hypothyroidism: a systematic review and meta-analysis. JAMA. 2018;320(13):1349-1359. doi:10.1001/jama.2018.13770 30285179 PMC6233842

[8] Jonklaas J, Bianco AC, Bauer AJ, ; American Thyroid Association Task Force on Thyroid Hormone Replacement Guidelines for the treatment of hypothyroidism: prepared by the american thyroid association task force on thyroid hormone replacement. Thyroid. 2014;24(12):1670-1751. doi:10.1089/thy.2014.0028 25266247 PMC4267409

[9] Pearce SH, Brabant G, Duntas LH, 2013 ETA Guideline: management of subclinical hypothyroidism. Eur Thyroid J. 2013;2(4):215-228. doi:10.1159/000356507 24783053 PMC3923601

[10] Rodriguez-Gutierrez R, Maraka S, Ospina NS, Montori VM, Brito JP Levothyroxine overuse: time for an about face? Lancet Diabetes Endocrinol. 2017;5(4):246-248. doi:10.1016/S2213-8587(16)30276-5 28029536

[11] Leading chemical drugs dispenses in England in 2019. Published 2020. Accessed July 10, 2020. https://www.statista.com/statistics/378445/prescription-cost-analysis-top-twenty-chemicals-by-items-in-england/

[12] Allport J, McCahon D, Hobbs FD, Roberts LM Why are GPs treating subclinical hypothyroidism? case note review and GP survey. Prim Health Care Res Dev. 2013;14(2):175-184. doi:10.1017/S1463423612000230 23174158

[13] Stott DJ, Gussekloo J, Kearney PM, Study protocol; thyroid hormone replacement for untreated older adults with subclinical hypothyroidism—a randomised placebo controlled trial (TRUST). BMC Endocr Disord. 2017;17(1):6. doi:10.1186/s12902-017-0156-8 28158982 PMC5291970

[14] The TRUST Study—Depression Substudy (TRUST). Clinicaltrials.gov identifier: NCT01853579. Updated June 14, 2018. Accessed January 4, 2021. https://clinicaltrials.gov/ct2/show/NCT01853579

[15] Schulz KF, Altman DG, Moher D; CONSORT Group CONSORT 2010 statement: updated guidelines for reporting parallel group randomised trials. BMJ. 2010;340:c332. doi:10.1136/bmj.c332 20332509 PMC2844940

[16] de Craen AJ, Heeren TJ, Gussekloo J Accuracy of the 15-item geriatric depression scale (GDS-15) in a community sample of the oldest old. Int J Geriatr Psychiatry. 2003;18(1):63-66. doi:10.1002/gps.773 12497557

[17] Sheikh JI, Yesavage JA, Brooks JO III, Proposed factor structure of the Geriatric Depression Scale. Int Psychogeriatr. 1991;3(1):23-28. doi:10.1017/S1041610291000480 1863703

[18] Vinkers DJ, Gussekloo J, Stek ML, Westendorp RG, Van Der Mast RC The 15-item Geriatric Depression Scale (GDS-15) detects changes in depressive symptoms after a major negative life event: the Leiden 85-plus Study. Int J Geriatr Psychiatry. 2004;19(1):80-84. doi:10.1002/gps.1043 14716703

[19] Sundermann EE, Katz MJ, Lipton RB Sex differences in the relationship between depressive symptoms and risk of amnestic mild cognitive impairment. Am J Geriatr Psychiatry. 2017;25(1):13-22. doi:10.1016/j.jagp.2016.08.022 27986237 PMC5215465

[20] Chwastiak L, Ehde DM, Gibbons LE, Sullivan M, Bowen JD, Kraft GH Depressive symptoms and severity of illness in multiple sclerosis: epidemiologic study of a large community sample. Am J Psychiatry. 2002;159(11):1862-1868. doi:10.1176/appi.ajp.159.11.1862 12411220

[21] Vogelzangs N, Beekman AT, Boelhouwer IG, Metabolic depression: a chronic depressive subtype? Findings from the InCHIANTI study of older persons. J Clin Psychiatry. 2011;72(5):598-604. doi:10.4088/JCP.10m06559 21535996 PMC6232848

[22] Morsink LF, Vogelzangs N, Nicklas BJ, ; Health ABC study Associations between sex steroid hormone levels and depressive symptoms in elderly men and women: results from the Health ABC study. Psychoneuroendocrinology. 2007;32(8-10):874-883. doi:10.1016/j.psyneuen.2007.06.009 17651906

[23] Vilagut G, Forero CG, Barbaglia G, Alonso J Screening for depression in the general population with the Center for Epidemiologic Studies Depression (CES-D): a systematic review with meta-analysis. PLoS One. 2016;11(5):e0155431. doi:10.1371/journal.pone.0155431 27182821 PMC4868329

[24] Imai H, Furukawa TA, Kasahara Y, Ipsative imputation for a 15-item Geriatric Depression Scale in community-dwelling elderly people. Psychogeriatrics. 2014;14(3):182-187. doi:10.1111/psyg.12060 25323959

[25] le Cessie S, Goeman JJ, Dekkers OM Who is afraid of non-normal data? choosing between parametric and non-parametric tests. Eur J Endocrinol. 2020;182(2):E1-E3. doi:10.1530/EJE-19-0922 31910149

[26] Altman NS An introduction to kernel and nearest-neighbor nonparametric regression. The American Statistician. 1992;46(3):175-185. doi:10.1080/00031305.1992.10475879

[27] Higgins JPT, Green S. Cochrane Handbook for Systematic Reviews of Interventions Version 5.1.0. Updated March 2011. Accessed July 7, 2020. https://handbook-5-1.cochrane.org/

[28] Schünemann H, Brożek J, Guyatt G, Oxman A, eds. GRADE Handbook. Updated October 2013 Accessed December 31, 2020. https://gdt.gradepro.org/app/handbook/handbook.html

[29] Jorde R, Waterloo K, Storhaug H, Nyrnes A, Sundsfjord J, Jenssen TG Neuropsychological function and symptoms in subjects with subclinical hypothyroidism and the effect of thyroxine treatment. J Clin Endocrinol Metab. 2006;91(1):145-153. doi:10.1210/jc.2005-1775 16263815

[30] Reuters VS, Almeida CdeP, Teixeira PdeF, Effects of subclinical hypothyroidism treatment on psychiatric symptoms, muscular complaints, and quality of life. Arq Bras Endocrinol Metabol. 2012;56(2):128-136. doi:10.1590/S0004-27302012000200006 22584566

[31] Najafi L, Malek M, Hadian A, Ebrahim Valojerdi A, Khamseh ME, Aghili R Depressive symptoms in patients with subclinical hypothyroidism—the effect of treatment with levothyroxine: a double-blind randomized clinical trial. Endocr Res. 2015;40(3):121-126. doi:10.3109/07435800.2014.896924 25775223

[32] Blum MR, Wijsman LW, Virgini VS, ; PROSPER study group Subclinical thyroid dysfunction and depressive symptoms among the elderly: a prospective cohort study. Neuroendocrinology. 2016;103(3-4):291-299. doi:10.1159/000437387 26202797

[33] Demartini B, Ranieri R, Masu A, Selle V, Scarone S, Gambini O Depressive symptoms and major depressive disorder in patients affected by subclinical hypothyroidism: a cross-sectional study. J Nerv Ment Dis. 2014;202(8):603-607. doi:10.1097/NMD.0000000000000168 25010109

[34] Grabe HJ, Völzke H, Lüdemann J, Mental and physical complaints in thyroid disorders in the general population. Acta Psychiatr Scand. 2005;112(4):286-293. doi:10.1111/j.1600-0447.2005.00586.x 16156836

[35] Gussekloo J, van Exel E, de Craen AJ, Meinders AE, Frölich M, Westendorp RG Thyroid status, disability and cognitive function, and survival in old age. JAMA. 2004;292(21):2591-2599. doi:10.1001/jama.292.21.2591 15572717

[36] Hong JW, Noh JH, Kim DJ Association between subclinical thyroid dysfunction and depressive symptoms in the Korean adult population: The 2014 Korea National Health and Nutrition Examination Survey. PLoS One. 2018;13(8):e0202258. doi:10.1371/journal.pone.0202258 30106989 PMC6091963

[37] Loh HH, Lim LL, Yee A, Loh HS Association between subclinical hypothyroidism and depression: an updated systematic review and meta-analysis. BMC Psychiatry. 2019;19(1):12. doi:10.1186/s12888-018-2006-2 30621645 PMC6325749

[38] Park YJ, Lee EJ, Lee YJ, Subclinical hypothyroidism (SCH) is not associated with metabolic derangement, cognitive impairment, depression or poor quality of life (QoL) in elderly subjects. Arch Gerontol Geriatr. 2010;50(3):e68-e73. doi:10.1016/j.archger.2009.05.015 19545916

[39] Wildisen L, Del Giovane C, Moutzouri E, An individual participant data analysis of prospective cohort studies on the association between subclinical thyroid dysfunction and depressive symptoms. Sci Rep. 2020;10(1):19111. doi:10.1038/s41598-020-75776-1 33154486 PMC7644764

[40] Parle J, Roberts L, Wilson S, A randomized controlled trial of the effect of thyroxine replacement on cognitive function in community-living elderly subjects with subclinical hypothyroidism: the Birmingham Elderly Thyroid study. J Clin Endocrinol Metab. 2010;95(8):3623-3632. doi:10.1210/jc.2009-2571 20501682

[41] Garber JR, Cobin RH, Gharib H, ; American Association of Clinical Endocrinologists and American Thyroid Association Taskforce on Hypothyroidism in Adults Clinical practice guidelines for hypothyroidism in adults: cosponsored by the American Association of Clinical Endocrinologists and the American Thyroid Association. Thyroid. 2012;22(12):1200-1235. doi:10.1089/thy.2012.0205 22954017

