# Supplementary material for: Effect of Levothyroxine Therapy on the Development of Depressive Symptoms in Older Adults With Subclinical Hypothyroidism: An Ancillary Study of a Randomized Clinical Trial
Source: JAMA Netw Open. 2021 Feb 10;4(2):e2036645. doi: 10.1001/jamanetworkopen.2020.36645 (PMC7876592; doi:10.1001/jamanetworkopen.2020.36645)
Supplement: Supplement 1. — Trial Protocol [file jamanetwopen-e2036645-s001.pdf]

Protocol version 6.1, 24th Mar

Multi-modal effects of Thyroid hormone Replacement for Untreated older adults with Subclinical hypothyroidism; a randomised placebo-controlled Trial

**SHORT TITLE**	TRUST
**Compound**	Levo-thyroxine T_4
**Version**	6.1
**Date**	24th March 2016
**EudraCT Number**	2011-004554-26

Co-Sponsors NHS Greater Glasgow and Clyde and The University of Glasgow

This study will have a sponsor for each participating Member / Associated State.

Funder European Commission

FP7-HEALTH-2011 (Seventh EU Framework Programme)/278148

This study will be performed according to the Research Governance Framework for Health and Community Care (Second edition, 2006) and The Medicines for Human Use (Clinical Trials) Regulations, 2004 SI 2004:1031 (as amended) and WORLD MEDICAL ASSOCIATION DECLARATION OF HELSINKI Ethical Principles for Medical Research Involving Human Subjects 1964 (as amended).

Contacts

Chief Investigator:
Professor David J Stott

Department of Academic Geriatric Medicine, University of Glasgow; Room 2.42
New Lister Building, Glasgow Royal Infirmary; Glasgow G31 2ER 4
Tel: +44 141 201 8510
Email: David.J.Stott@glasgow.ac.uk

Co-investigators: Professor Naveed Sattar
Professor of Metabolic Medicine, Wolfson Medical School Building,
University Avenue, Glasgow, G12 8QQ, Scotland
Tel: +44 141 330 3419
Email:naveed.sattar@glasgow.ac.uk

Dr Terry J Quinn
Department of Academic Geriatric Medicine, University of Glasgow; Room 2.44
New Lister Building, Glasgow Royal Infirmary, Glasgow G431 2ER, Scotland
Tel: 0141 2018510
Email: tjq1t@clinmed.gla.ac.uk

Professor Ian Ford
Robertson Centre for Biostatistics, University of Glasgow, Level 11 Boyd Orr Building, Glasgow, G12 8QQ
Tel: 0141 330 4744
Email: ian.ford@glasgow.ac.uk

Ms Liz Anderson
Robertson Centre for Biostatistics, University of Glasgow, Level 11 Boyd Orr Building, Glasgow, G12 8QQ
Tel: 0141 330 6279
Email: liz.anderson@glasgow.ac.uk

Ms Sharon Kean
Robertson Centre for Biostatistics, University of Glasgow, Level 11 Boyd Orr Building, Glasgow, G12 8QQ
Tel: 0141 330 3266
Email: sharon.kean@glasgow.ac.uk

Dr Patricia Kearney
Senior Lecturer, University College Cork, National University of Ireland, Cork,
Tel: +353 21 4901592
Email: patricia.kearney@ucc.ie

Professor Jacobijn Gussekloo
Department of Public Health and Primary Care, Leiden University Medical Centre,
Leiden, 2333 ZA, Netherlands
Tel: +3171526 84444
Email: J.Gussekloo@lumc.nl

Protocol version 6.1, 24th Mar

Professor Nicolas Rodondi
University of Bern, 4 Hochschulstrasse, BERN, 3012, Switzerland
Tel: +41 31 632 2525
Email: Nicolas.Rodondi@hospvd.ch

Professor Rudi G J Westendorp
Leyden Academy on Vitality and Ageing, 10 Rijnsburgerweg Poortgb K, Leiden, 2333 AA, Netherlands
Tel: +31715240960
Email: westendorp@leydenacademy.nl

Trial Statistician:
Professor Ian Ford
Robertson Centre for Biostatistics, University of Glasgow, Level 11 Boyd Orr Building, Glasgow, G12 8QQ
Tel: 0141 330 4744
Email: ian.ford@glasgow.ac.uk

Project Manager/Study Co-ordinator:
Mairi McDade
Department of Academic Geriatric Medicine, University of Glasgow; Room 2.45 New Lister Building, Glasgow Royal Infirmary; Glasgow G31 2ER
Tel: +44 141 201 8522
Email: mairi.mcdade@glasgow.ac.uk

Pharmacy:
Dr Elizabeth Douglas

Clinical Trials Pharmacist, Research & Development, NHS Greater Glasgow & Clyde, West Glasgow Ambulatory Care Hospital, Dalnair Street,Glasgow G3 8SW 1st Floor
Tel: +44 141 2232 1792
Email: elizabeth.douglas@ggc.scot.nhs.uk

Pharmacovigilance: Pharmacovigilance Office
Glasgow Clinical Trial’s Unit, Robertson Centre for Biostatistics, 11th Floor, Boyd Orr Building, University of Glasgow, University Avenue, Glasgow, G12 8QQ.
Tel: 0141 3304744
Fax: 0141 3575588

Sponsor’s contact:
Paul Dearie
Research & Development, NHS Greater Glasgow and Clyde, 1st Floor, R&D Central Office, West Glasgow Ambulatory Care Hospital, Dalnair Street, Glasgow, G3 8SW
Tel: +141 232 1820
Email paul.dearie@ggc.scot.nhs.uk

Funder:
European Commission

Seventh Programme Framework. Specific Programme “Cooperation” – Theme "Health" Call identifier: FP7HEALTH2011twostage

Clinical laboratories, medical or technical departments or institutions involved in the trial:
Clinical biochemistry laboratories, NHS Greater Glasgow and Clyde

Protocol version 6.1, 24th Mar

Protocol Approval

TRUST - Multi-modal effects of Thyroid hormone Replacement for Untreated older adults with Subclinical hypothyroidism; a randomised placebo-controlled Trial

Chief Investigator: Professor David J Stott

Address: Department of Academic Geriatric Medicine, University of Glasgow
Walton Building,
Glasgow Royal Infirmary; Glasgow, G4 0SF

Signature:

Date: 24th Mar 2016

Sponsor Representative: Dr Paul Dearie

Address: NHS Greater Glasgow and Clyde, Clinical Research & Development,
West Glasgow Ambulatory Care Hospital
Dalnair Street
Glasgow, G3 8SW.

Signature:

Date: 24th March 2016

University of Glasgow

NHS Greater Glasgow and Clyde

SEVENTH FRAMEWORK PROGRAMME

Protocol version 6.1, 24th Mar

TABLE OF CONTENTS

	ABBREVIATIONS	7
	STUDY SYNOPSIS	8
	SCHEDULE OF ASSESSMENTS	10
1.1	INTRODUCTION	11
1.2	Background	11
1.3	Study Rationale - Hypothesis	12
2.1	STUDY OBJECTIVES	13
3.1	STUDY DESIGN	14
3.2	Study Population	14
3.3	Main Inclusion Criteria	14
3.4	Main Exclusion Criteria	14
3.5	Identification of Participants and Informed Consent	15
3.6	Withdrawal	17
3.7	Blinding	17
4.1	MEDICATIONS/TREATMENTS	17
4.2	Levothyroxine	18
4.2.1	Side Effects	18
4.2.2	Pharmacodynamic properties	18
4.2.3	Pharmacokinetic properties	18
4.2.4	Rationale for chosen drug	18
4.3	Study Intervention	19
4.4	Formulation and Source of Drug	20
4.5	Storage and Stability	20
4.6	Drug Procurement	20
4.6.1	Packing and Distribution	21
4.6.2	Drug Accountability	21
4.7	Destruction of Unused Drug	21
4.8	Unblinding of Treatment Allocation	21
4.9	Criteria for Withdrawal of Participants on Safety Grounds and Withdrawal	21
4.10	Maintenance of trial treatment randomisation codes and procedures for unblinding.	22
4.11	Special warnings and precautions for use	22
4.12	Interactions with other drugs	22
5.1	METHODS OF DATA COLLECTION	24
5.2	Data Collection	25
5.3	Data Collection Process	26
6.1	ASSESSMENT AND REPORTING OF ADVERSE EVENTS/SERIOUS ADVERSE EVENTS	27
6.2	Definitions of Adverse Events	27
6.3	Definitions of Serious Adverse Event (SAE) or Serious Adverse Reaction (SAR)	27
6.4	Definition of Suspected Serious Adverse Reaction (SSAR)	28
6.5	Definition of Suspected Unexpected Serious Adverse Reaction (SUSAR)	28
6.6	Recording and Reporting AEs/SAEs	28
6.7	Unblinding	30
6.8	Specific adverse events of interest	30
6.9	Annual Safety Report	30
6.10	Safety Flow Chart	31
7.0	Assessment and follow-up	32

8.1 STATISTICAL ANALYSIS 33
8.2 Statistical Analysis Plan 33
8.3 Pilot data, power calculations and justification of planned sample size 34
8.4 Revised Power calculations and sample size 35
8.5 Statistical Methods
9 END OF TRIAL 35
10 TRIAL MANAGEMENT 36
10.1 Study administrative team 36
10.2 Trial Steering Committee 36
10.3 Independent Data and Safety Monitoring Committee (IDMC) 37
10.4 National organising committees 37
11 CO-SPONSOR RESPONSIBILITIES (NHS GREATER GLASGOW AND CLYDE/UNIVERSITY OF GLASGOW) 37
11.1 Study monitoring and auditing 38
11.2 Protocol amendments 38
12 INDEMNITY AND INSURANCE 38
13 FUNDING 39
14 PUBLICATION 39
15 ARCHIVING 40
16 REFERENCES 41
APPENDICES 44

Appendix 1: Outcome scales 45

Addendum Revised projected recruitment numbers and implications for conduct and statistical power of the study

35

Protocol version 6.1, 24th Mar

ABBREVIATIONS

AE	Adverse Event
AR	Adverse Reaction
AD	Activities of daily
L	living Atrial
AF	fibrillation
BI	Barthel Index
BMI	Body mass index
CH	Coronary heart disease
D CI	Chief Investigator
CRF	Case report form
EC	Ethics Committee
eCRF	Electronic case report form
ECG	Electrocardiogram
GP	General Practitioner
HR	Hazard ratio
IB	Investigator brochure
ICH GCP	International Conference on Harmonization of Good Clinical Practice
IDM	Independent data monitoring committee
C	Letter-Digit Coding Test
LDC	Mini mental state
T	New York Heart Association
MMSE	Older American resources and services measure of activities of daily living
NYH	Principal investigator
A	Prospective study of pravastatin in the elderly at risk
OAR	Robertson Centre for Biostatistics
S PI	Randomised controlled trial
PROSPER	Serious Adverse Event
RCB	Statistical Analysis Plan
RC	Serious Adverse Reaction
T	Serious Suspected Adverse Reaction
SAE	
SAP	
SAR	
SSAR	
SUSAR	Suspected Unexpected Serious Adverse Reaction
SCH	Subclinical hypothyroidism
SOP	Standard operating procedure
T_4	Levothyroxine
	Thyroid disease quality of life
ThyDQo	Thyroid symptom questionnaire
L	Thyroid stimulating hormone
ThySR	Upper limit of normal
Q TSH	Quality of life
ULN	
QOL	

Protocol version 6.1, 24th Mar

STUDY SYNOPSIS

Title of Study:	**TRUST**<br>Multi-modal effects of Thyroid hormone Replacement for Untreated older adults with Subclinical hypothyroidism; a randomised placebo-controlled Trial
Study Centres:	NHS Greater Glasgow & Clyde<br>University College Cork<br>Leiden University Medical Centre<br>University of Bern<br>Leiden Academy on Vitality and Ageing
Data Centre	Robertson Centre for Biostatistics, University of Glasgow
Duration of Study:	4 years
Objectives:	To test the efficacy of thyroxine replacement for subclinical hypothyroidism (SCH) in older adults
Primary Objective:	To determine multi-modal effects (cognitive; musculoskeletal and quality of life) of levo-thyroxine treatment for SCH in older adults
Secondary Objectives:	1) To determine effects of SCH treatment in various subgroups<br>2) To determine adverse effects associated with SCH treatment with particular focus on arrhythmia and heart failure<br>3) To establish a blood bio-bank, to be used in future research into causes and mechanisms of health, disease and disability in later life (this is not directly funded through this research application)
Study Endpoints	1) Fatal and non-fatal cardiovascular events<br>2) Change in disease specific QOL and symptom burden<br>3) General QOL<br>4) Handgrip strength<br>5) Cognitive function<br>6) Total mortality<br>7) Functional ability (basic and extended activities of daily living)
Rationale:	To provide the necessary evidence to properly inform best practice for treatment of SCH in older people
Methodology:	Randomised double-blind placebo-controlled parallel group trial of Levothyroxine for older people with subclinical hypothyroidism
Sample Size:	540 to 750 people
Screening	Potential subjects will be identified from clinical laboratory databases as having biochemical features consistent with SCH, (Thyroid stimulating hormone [TSH] of ≥4.6 and ≤19.9 mU/L plus free thyroxine levels within the laboratory reference range)
Registration/Randomisation:	Randomisation (1:1 Levothyroxine versus placebo) will

	be stratified by site, gender and starting dose of levo-thyroxine, and carried out using the method of randomly permuted blocks
Inclusion Criteria	Community-dwelling subjects aged ≥65 years with SCH, diagnosed on the basis of elevated TSH plus free thyroxine within the laboratory reference range, measured on a minimum of two occasions at least 3 months apart
Exclusion Criteria	• Subjects currently on (anti)thyroid drugs, amiodarone or lithium<br>• Recent thyroid surgery or radio-iodine<br>• Grade IV NYHA heart failure<br>• Prior clinical diagnosis of dementia<br>• Recent hospitalisation for major illness<br>• Recent acute coronary syndrome<br>• Acute myocarditis or acute pancarditis<br>• Untreated adrenal insufficiency or adrenal disorder<br>• Terminal illness<br>•Patients with rare hereditary problems of galactose intolerance, the Lapp lactase deficiency or glucose-galactose malabsorption<br>• Subjects who are participating in ongoing RCTs of therapeutic interventions (including CTIMPs)<br>• Plan to move out of the region in which the trial is being conducted within the next 2 years
Product, Dose, Modes of Administration:	Oral Levothyroxine starting dose 50 µg daily (reduced to 25 µg daily in subjects <50Kg body weight, or if known coronary heart disease) versus matching placebo.<br>The dose will be changed according to the serum TSH level measured at 6-8 weeks after starting medication and after each dose change. Dose titration will be according to a predefined dosing schedule
Duration of Treatment:	Minimum 1 year
Statistical Analysis	To include, time to first event Cox regression analysis stratified by gender in models containing the randomised treatment allocation as a covariate (intention-to-treat).<br>Tests of treatment effect will be based on the Wald test and corresponding point estimates and 95% confidence intervals for the hazard ratio for treatment will be calculated. The assumption of proportionality of hazards will be tested.

SCHEDULE OF ASSESSMENTS

(*the final visit assessments may substitute for any assessment time between 12 and a maximum of 42 months).

Weeks (wks) or Months (m) of follow up

	**Screen** visit	**0** visit	**6-8wks** visit	**6m** call	**12m** visit	**18m** call	**24m** visit	**30m** call	**36m** visit	**Final*** visit
Age and gender	x									
Medical history	x									
Concomitant medication	x		x		x		x		x	x
**Safety and monitoring**										
Morbidity, mortality			x	x	x	x	x	x	x	x
SAEs			x	x	x	x	x	x	x	x
Single-lead ECG (for AF)		x			x		x		x	x
Drug adherence			x		x		x		x	x
**Outcomes**										
Cardiovascular events			x	x	x	x	x	x	x	x
Quality of life measures		x	x		x					x
Grip strength		x			x					x
Cognitive function										
MMSE		x								
Letter-Digit Coding Test		x								x
Blood pressure	x				x					x
Functional status										
ADL; IADL		x								x
Home support		x								x
Institutionalisation		x								x
**Laboratory analysis**										
Thyroid function	x		x		x		x		x	x
Full Blood Count		x			x					
Biobank samples		x			x					

†Thyroid function will be checked at 6-8 weeks after dose titrations as per protocol.

Interim visits 12m, 24m, 36m may be performed +/- 1 month.

1.1 INTRODUCTION

1.2 Background

Subclinical hypothyroidism (SCH) is a common finding in older people across Europe. It is defined as an elevated serum thyroid-stimulating hormone (TSH) with normal circulating thyroid hormone levels (1). The prevalence is around 8% in adult women and 3% in men, but the proportion of the population affected rises markedly with increasing age. Approximately 8-18% of adults over 65 years have SCH, prevalence being higher among women (2-4). SCH is a likely contributor to multiple problems in older age. From a biological point of view thyroid hormone has multiple pleiotropic effects, acting as an essential regulatory factor in numerous physiological systems, including the vascular tree and the heart, brain (including cognition and mood), skeletal muscle and bone. Health consequences of overt thyroid disease range from mild non-specific symptoms such as tiredness (5) that can adversely affect quality of life, to coronary heart disease (CHD).

There are data to suggest adverse health effects of SCH. The most convincing epidemiological associations of SCH with poor health are with coronary heart disease (CHD) events and deaths. We performed an individual patient data analysis from over 50,000 subjects in 11 prospective cohorts (2); the age- and sex-adjusted hazard ratio (HR) for CHD mortality was 1.09 (95% CI 0.91-1.30) for a TSH of 4.5-6.9mU/L, 1.42 (1.03-1.95) for a TSH of 7.0-9.9 mU/L and 1.58 (1.10-2.27) for a TSH of 10.0-19.9mU/L; corresponding HRs for CHD events were 1.00 (0.86-1.18), 1.17 (0.96-1.43) and 1.89 (1.28-2.80) respectively. In contrast to the epidemiological associations found in the whole population, we have found that SCH in advanced older age (>85 yrs) might be associated with better health and survival compared to the euthyroid state (6), giving rise to the possibility that there may be an age interaction for thyroid hormone effect.

There is considerable symptom burden in hypothyroid states. Patients with overt hypothyroidism are most bothered by hair problems, weight gain, depression, fatigue and feeling cold. Of these symptoms, depression, feeling cold, and tiredness are significantly correlated with their ratings of poorer present quality of life (7). By definition, subjects with subclinical hypothyroidism do not have the full symptom cluster of overt hypothyroidism, however they often report non-specific symptoms such as tiredness (5). Health-related quality of life (as measured by the Short Form-36 questionnaire) is reduced in subjects with SCH compared to euthyroid controls, but there is a spectrum with worst status in subjects with overt hypothyroidism (8). Muscle symptoms such as cramps, weakness and myalgia are more common in SCH than in euthyroid controls (9). SCH has also been linked with adverse psychiatric outcomes (including cognition and mood disorder (10)) although data are

inconsistent (11) and associations are less strong than for cardiovascular disease. Reduced exercise capacity in SCH may be due to impaired skeletal muscle function (9) and increased oxygen requirements of exercise (12). SCH has also been associated with systolic and diastolic cardiac dysfunction and with an increased incidence of clinical heart failure (13). Subclinical hypothyroidism may also cause a low-grade anaemia; in a large observational study, in euthyroid participants, each 1.0 pM increase in free T4 was associated with an increase in haemoglobin of 0.39 g/l (26). These adverse effects are likely to reduce maximal exercise capacity, and might have important population effects on abilities to perform activities of daily living, although reduced functional capacity in SCH has not been demonstrated in epidemiological studies (14).

Evidence is lacking about the benefits of Levothyroxine replacement in the elderly with SCH, as no large randomized clinical trials (RCT) on the full range of relevant clinical outcomes have been performed (3). The indications for screening and threshold TSH for treatment of SCH are areas of clinical controversy. The Cochrane systematic review of Levothyroxine replacement for SCH summarises the evidence from RCTs up to 2006 (3). It concluded that there was some evidence for improved cardiac function and blood lipids with Levothyroxine replacement, but a lack of data for improved survival, reduced cardiovascular morbidity or improved health-related quality of life; data were available for only 350 patients in twelve RCTs, often of short duration (range 6-14 months).

Thus there is the potential for multisystem benefits from treatment of SCH with Levothyroxine. The high prevalence of SCH in later life gives the prospect that the population attributable benefit of treatment could be large. However definitive evidence from RCTs is lacking.

1.3 Study Rationale - Hypothesis

There is reason to believe that treatment of SCH in older adults may have multi-modal health benefits, however definitive trial evidence is lacking. Strong recommendation was made in the Cochrane review of 2007 (3), by international experts (15) and by the US Preventive Services Task Force (16) for further RCTs in larger groups and with longer follow-up for clinical endpoints.

To definitively solve this clinical uncertainty, we propose to conduct the first large RCT with power to detect clinically worthwhile benefits from Levothyroxine replacement for SCH. Critical elements of the study design include longer follow-up than previous RCTs, recruitment of subjects with persisting SCH (excluding those in whom it is a temporary phenomenon, who are less likely to benefit), and clinically important outcomes. We will recruit subjects with a wide range of characteristics (age, gender, TSH levels) to allow pre-planned subgroup analysis and potential targeting of treatment to subjects with specific characteristics. We also propose a

wide range of outcome assessments reflecting the potential multi-system effects of Levothyroxine replacement for SCH.

We propose a randomised double-blind placebo-controlled parallel group trial of Levothyroxine for older people with SCH. We propose a pragmatic study that will generate data that are generalisable and therefore will directly influence clinical practice. This includes a study population with wide age range and no upper limits, a target TSH within the laboratory reference range (reflecting common clinical practice) and simple inclusion criteria (for example not including thyroid auto-antibodies). We have selected a wide range of clinical endpoints to reflect the potential multi-system and multi-modal effects of thyroid hormone. The study will conform to CONSORT guidelines (www.consort-statement.org).

2.1 STUDY OBJECTIVES

There are four main study objectives:

1. Does Levothyroxine treatment for SCH give multi-modal benefits for older people with SCH?

2. Are benefits seen across a wide range of outcomes, including prevention of cardiovascular disease, and improving health-related quality of life, muscle function and cognition?

3. Are benefits seen in specific subgroups of older people with SCH, including women, very elderly and those with mild degrees of SCH (TSH 4.6-10 mU/L)?

4. Are any benefits offset by adverse effects, such as atrial fibrillation or heart failure?

Additional study objective:
To establish a blood bio-bank, to be used in future research into causes and mechanisms of health, disease and disability in later life (this is not directly funded through this research application). The biobank will be maintained by NHS Greater Glasgow and Clyde and will be managed in line with all applicable and current regulations.

Primary endpoints:

(1) Change in disease specific QOL (measured using symptom and fatigue domains from the Thyroid-specific Quality of Life patient-reported outcome measure (ThyPRO) – measured at baseline; 6-8 weeks; 12 months and close-out.

Secondary endpoints:

(1) General QOL (measured using EuroQOL) at baseline; 6-8 weeks; 12 months and final follow up.
(2) Comprehensive thyroid quality of life assessment ThyPRO39 - recorded at final follow-up (additional 28 questions)
(3) Handgrip strength (measured using the Jadaar hand dynamometer) at baseline; 12 month and final follow up.
(4) Cognitive function, particularly executive function (measured using Letter Digit Coding Test [LDCT) at baseline and final follow-up.
(5) Fatal and non-fatal cardiovascular events (this will include acute myocardial infarction; stroke; amputations for peripheral vascular disease;

revascularisations for atherosclerotic vascular disease, including for acute coronary syndrome and heart failure hospitalisations).

(6) Total mortality and cardiovascular mortality
(7) Functional ability (basic Activities of Daily Living (ADL) measured using Barthel Index [BI]; extended activities of daily living measured using the older American resources and services [OARS]) at baseline and final follow-up.
(8) Haemoglobin, measured on a full blood count at baseline and 1 year.
(9) Blood pressure, measured at screening, 1 year and at final review.
(10) Weight and waist circumference, measured at screening, 1 year and/or at final review.

3.1 Study Design

This is a randomised double-blind placebo-controlled parallel group trial of Levothyroxine for older people with subclinical hypothyroidism. The trial will run over four years across four international sites (Glasgow, Cork, Leiden and Bern). We propose a minimum 1 year of follow-up, with a likely average of 3 years.

3.2 Study Population

The trial will recruit 540 to 750 community-dwelling patients aged ≥65 years with SCH, diagnosed on the basis of persistently elevated TSH levels, measured on a minimum of two occasions at least 3 months apart, over 2 years. Potential subjects will be identified from clinical laboratory databases as having biochemical features consistent with SCH.

We have defined SCH as persistently elevated TSH levels (≥4.6 and ≤19.9 mU/L) and free thyroxine (fT4) in normal range measured on a minimum of two occasions at least 3 months apart. Given the epidemiology of SCH we anticipate around two-thirds of subjects will be female.

Due to significantly lower than expected recruitment rates at all study sites, it is expected that substantially fewer subjects than planned will be recruited into the study. See addendum p57 for revised recruitment numbers and discussion on revised primary and secondary end-points.

3.3 Main Inclusion Criteria

Community-dwelling patients aged ≥65 years with SCH.

SCH is defined as persistently elevated TSH levels ($\geq$4.6 and $\leq$19.9 mU/L) and free thyroxine (fT4) in reference range measured on a minimum of two occasions at least 3 months apart.

3.4 Main Exclusion Criteria

- Subjects currently on Levothyroxine, antithyroid drugs, amiodarone or lithium.
- Recent thyroid surgery or radio-iodine (within 12 months).
- Grade IV NYHA heart failure.
- Prior clinical diagnosis of dementia.
- Recent hospitalisation for major illness or elective surgery (within 4 weeks).
- Recent acute coronary syndrome, including myocardial infarction or unstable angina (within 4 weeks).
- Acute myocarditis or acute pancarditis.
- Untreated adrenal insufficiency or adrenal disorder.
- Terminal illness.
- Patients with rare hereditary problems of galactose intolerance, the Lapp lactase deficiency or glucose-galactose malabsorption.
- Subjects who are participating in ongoing RCTs of therapeutic interventions (including CTIMPs)
- Plan to move out of the region in which the trial is being conducted within the next 2 years.

Atrial fibrillation (sustained or paroxysmal) will not be an exclusion, as in itself this cardiac arrhythmia is not a contra-indication to Levothyroxine treatment. In addition AF is a common finding in the studied age groups and exclusion of subjects with it would potentially compromise the generalisability of our results.

Adherence to treatment allocation: drop-ins (where subjects allocated to placebo are prescribed Levothyroxine) and drop outs (where subjects allocated to Levothyroxine stop this treatment) are each estimated at less than 5% at 1 year and less than 10% at the end of the study.

3.5 Identification of Participants and Informed Consent

Potential participants will be identified from clinical laboratory databases as having, within the last 36 months, biochemical features consistent with SCH with a TSH level $\geq$4.6 and $\leq$19.9 mU/L and age $\geq$65 years. There will be a minimum delay of 3 months between the last measure of TSH and the screening visit.

The initial laboratory identification of potentially eligible subjects will be for the previous 36 months, with subsequent new summary laboratory reports of potential cases at 3 monthly intervals (at 15, 18 and 21 months after commencement of the randomised controlled trial), allowing inclusion of 4 years of laboratory data for study recruitment.

The clinical laboratory will forward laboratory results, patient name and CHI number, and GP contact details to a safe haven at NHS Greater Glasgow and Clyde. This can be used to generate a standard letter to GPs for each potentially eligible patient, including a simple questionnaire indicating whether there are any reasons that the patient should not be invited for participation in the study, using information from their medical records, plus, if appropriate an invitation letter and information sheet.(with freephone telephone number) to be sent out by the GP to the patient, inviting them to attend a screening clinic (for thyroid function testing) The patient will be asked to indicate their willingness to be considered for the study by either returning a tick-box slip (freepost) or by telephoning the freephone line. For those unable to attend, or if it is the patient's preference we will offer review within their own home (arranged by patient by freephone call). For those who do not respond to the initial invitation for the screening visit, the GP will be sent a further invitation letter and information sheet to post to the patient (if appropriate).

Research sites out with NHS Greater Glasgow and Clyde that may not have an established link with NHS Greater Glasgow and Clyde Safe haven, General Practices will be approached for permission for screening of their records.

It is anticipated that most patients will be identified through thyroid function tests checked in primary care, however some will be identified through secondary care. When this is the case, the secondary care physician may act as the gatekeeper, indicating whether the patient is suitable for study entry and forwarding the invitation to indicate willingness for screening directly to the patient. If a subject is identified and recruited through secondary care the GP will be informed of their entry to the study.
To improve dissemination of information about the study, and to alert GPs to the possibility that they may have patients who are eligible for participation, the laboratories will generate an automated comment to be added to selected suitable blood results going to the GP as follows – 'Results of TFT tests suggest that this patient may be suitable for enrolment in the TRUST study. Information on the study is available from the TRUST team at (0141 201 8522)'. The laboratory computer system will generate this if TSH 4.6-19.9 and fT4 in reference range, patient is over 65, and not noted to be on thyroxine (tick box on request form). This notification does not require the study team to look at confidential data (it is generated automatically) and should the GP wish to take up the offer we then proceed to involve their general practice and their patient according to study protocol and ethical approvals.

Information sheets and consent forms for UK patients will be in the English language. Full written contact details and free-phone telephone support will be available for

screenees and participating patients, which they will be encouraged to use if they have concerns or questions about the study.

The time between screening (and provision of information sheets) and signing of formal consent for participation in the randomised controlled trial (prior to study baseline assessments) will allow ample opportunity for prospective entrants to reflect on participation and consider / ask further questions (free-phone contact provided). There will be no time or other pressure to give informed consent.

Participants will have the right:

- To know that participation is voluntary
- To ask questions and receive understandable answers before making a decision.
- To know the degree of risk and burden involved in participation
- To know who will benefit from participation
- To know the procedures that will be implemented in the case of incidental findings
- To receive assurances that appropriate insurance cover is in place
- To know how their data will be collected, protected during the project and either destroyed or reused a the end of the research, if plan to reuse the data exist, participants should be duly informed, and consented also for this further usage,
- To withdraw themselves and their data from the project at any time
- To know of any potential commercial exploitation of the research

At the screening visit, written informed consent for screening will be obtained, eligibility criteria will be reviewed, and a venous blood sample for baseline thyroid function tests (TSH and fT4) by a research nurse who will explain the study procedures including providing and information sheets on the randomised controlled trial. Consent will be taken by the study research nurse.

Those eligible subjects found to have both a TSH level of ≥4.6 and ≤19.9 mU/L and fT4 in the laboratory reference range at the screening visit will be assumed to have persistent biochemical SCH. They will be sent a written invitation or a telephone call from the nurse to take part in the study with a suggested date and time for a baseline study visit. Subjects who wish to decline to take part or find the offered date unsuitable will be advised to make a telephone call to the national study centre freephone to inform the study team or make alternative arrangements as appropriate. For those unable to attend, or if it is the patient's preference we will offer review within their own home (arranged by patient by freephone call as above). Subjects whose repeat screening thyroid function tests show TSH has reverted to within the normal range will be sent an information letter advising them that the results are normal and that it would not be appropriate for them to enter a trial of thyroid hormone treatment. This letter will be copied to the GP. Subjects whose repeat screening thyroid function tests show high TSH and low fT4 will be sent an information letter advising them that their thyroid gland appears to be underactive and that they should discuss need for treatment with their own doctor.

3.6 Withdrawal

The participant can decide to withdraw from the study at any time. The researcher also has the right to withdraw participants from the study is he/she feels that it is in the best interests of the participant. Full details of the reasons for withdrawal should be recorded on the CRF. Withdrawn participants should be followed up in accordance with the protocol. If a patient withdraws consent from treatment and from follow-up this should be clearly documented in the CRF.

3.7 Blinding

The study will be double blinded. Subject blinding to treatment allocation will be ensured through use of matched tablets for Levothyroxine and placebo. Clinicians / study centres blinding to treatment allocation will be ensured by remote laboratory analysis of blood samples for TSH with corresponding titration advice.

All blood tests for in-study thyroid stimulating hormone (TSH) and free thyroxine (fT4) levels will be performed by the research team. All TSH and fT4 results from the follow-up phase of the study will be returned directly to the data-centre, who will advise the clinical research team on any dose titration. The clinical research team will not be informed of the actual results of thyroid function testing. The same process of blinding will be followed for measurement of haemoglobin on the full blood count. Detailed algorithms for titration of Levothyroxine and placebo, including dosing of Levothyroxine and numbers of tablets to be consumed daily, will be prepared in the initial planning and implementation phase of the study.

These processes are designed to ensure the integrity of blinding, with the research and the clinical teams kept unaware of results of repeat thyroid function tests. Drug and placebo supplies will be either provided or posted to patients at visit 0 and will then be posted out to patients after each check of thyroid function and at interim time-points of 6, 18 and 30 months; a process for tracking receipt of study medication will be used.

During the TRUST study there will be risk of unblinding through additional unscheduled GP or hospital testing of thyroid function. We intend to minimise the risk of this happening by effective communication, including through the study website and freephone access for patients and their physicians. In this communication we will discourage the practice of unnecessary interim testing of thyroid function.

4.1 Levothyroxine

The investigational medicinal product will be Levothyroxine (T4) as tablets for oral administration. Oral Levothyroxine is widely used as the sole treatment for overt hypothyroidism and is the obvious intervention to trial for SCH. The main possible alternative (or additional treatment) is tri-iodothyronine, however this short-acting hormone is less tried and tested and is likely to carry increased risk of adverse effects (particularly with over-replacement). It is therefore not an attractive option. Tablets will contain Levothyroxine Sodium also known as thyroxine sodium. Matching placebo will also be produced.

4.1.1 Side Effects

Side-effects are usually indicative of excessive dosage and usually disappear on reduction of dosage or withdrawal of treatment for a few days. Such effects include:
General: Headache, flushing, fever and sweating
Immune system disorders: hypersensitivity reactions including rash, pruritus oedema, dyspnea, joint pain and malaise
Metabolic: weight loss
Nervous system: tremor, restlessness, excitability, insomnia. Cardiac: anginal pain, cardiac arrythmias, palpitations, tachycardia
Gastrointestinal: diarrhoea, vomiting
Musculoskeletal and connective tissue: muscle cramps, muscle weakness. Reproductive: menstrual irregularities
Heat intolerance, transient hair loss in children.
Some patients may experience a severe reaction to high levels of thyroid hormone. This is called a 'thyroid crisis' with any of the following symptoms; hyperpyrexia, tachycardia, arrhythmia, hypotension, cardiac failure, jaundice, confusion, seizure and coma.

4.1.2 Pharmacodynamic properties

Levothyroxine is deiodinated in peripheral tissues to form triiodothyronine which is thought to be the active tissue form of thyroid hormone. Triiodothyronine has a rapid action but a shorter duration of activity than Levothyroxine. The chief action of Levothyroxine is to increase the rate of cell metabolism.

4.1.3 Pharmacokinetic properties

Levothyroxine sodium is incompletely and variably absorbed from the gastrointestinal tract. It is almost completely bound to plasma proteins and has a half-life in the circulation of about a week in healthy subjects, but longer in patients with myxoedema.
A large portion of the Levothyroxine leaving the circulation is taken up by the liver. Part of a dose of Levothyroxine is metabolised to triiodothyronine. Levothyroxine is excreted in the urine as free drug, deiodinated metabolites and conjugates. Some Levothyroxine is excreted in the faeces.

4.1.4 Rationale for chosen drug

Oral Levothyroxine is widely used as the sole treatment for overt hypothyroidism and is the obvious intervention to trial for subclinical hypothyroidism. The main possible alternative (or additional treatment) is tri-iodothyronine, however this short-acting hormone is less tried and tested and is likely to carry increased risk of adverse effects (particularly with over-replacement). It is therefore not an attractive option.

4.2 Study Intervention

The intervention will start with Levothyroxine 50 micrograms daily (reduced to 25 micrograms in subjects <50Kg body weight or if known coronary heart disease – previous myocardial infarction or symptoms of angina pectoris) versus matching placebo for 6-8 weeks.

The dose will be changed according to the serum TSH level as follows; at 6-8 weeks a blood sample will be taken for serum TSH, with 3 possible actions;

1) TSH <0.4 mU/L; treatment dose reduced to 25 micrograms Levothyroxine in those starting on 50 micrograms; reduced to 0 in those starting on 25 micrograms – effected by giving placebo matching the 25 micrograms dose; these patients will have a further check TSH after 6-8 weeks; if TSH remains <0.4 mU/L patient will be withdrawn from randomised treatment.
2) TSH ≥0.4 and <4.6 mU/L; no change to the treatment dose; patient to be reviewed at 12 months.
3) TSH remains elevated (≥4.6mU/L); additional 25 micrograms Levothyroxine, giving a total daily dose of 75 micrograms Levothyroxine for those starting on 50 micrograms, or a total daily dose of 50 micrograms Levothyroxine for those starting on 25 micrograms; further check TSH after 6-8 weeks repeating this cycle one more time; if TSH <0.4 mU/L; treatment dose reduced by 25 micrograms, with further repeat at 6-8 weeks as per 1) above. If TSH remains elevated elevated (≥4.6mU/L); additional 25 micrograms Levothyroxine, giving a total daily dose of 100 micrograms Levothyroxine for those starting on 50 micrograms, or a total daily dose of 75 micrograms Levothyroxine for those starting on 25 micrograms; after these dose changes a further check TSH will be performed after 6-8 weeks, and if TSH <0.4 mU/L; treatment dose reduced by 25 micrograms, with further repeat at 6-8 weeks as above. This strategy is designed to avoid over-replacement of levothyroxine.

The above process (but with only a single up-titration) will be repeated at 12 months (plus or minus 1 month) then annually (at 24 and 36 months plus or minus 1 month) with further dose increments of 25 micrograms if TSH elevated (≥4.6 mU/L), and 25 micrograms dose reductions if TSH suppressed (<0.4 mU/L). For all patients who have a dose increase (including at annual review) further check TSH will be performed after 6-8 weeks, and if TSH <0.4 mU/L; treatment dose reduced by 25 micrograms, with further repeat at 6-8 weeks as above. If TSH remains <0.4 mU/L on 2 consecutive measurements (6-8 weeks apart) the patient will be withdrawn from randomised treatment.

Therefore in summary there will be a maximum of 2 up-titrations of dose at the start of the study (each at 6-8 week intervals), and a maximum of only 1 up-titration at each annual review. If a patient is found to have a suppressed TSH level, the dose of levothyroxine will be reduced by 25 microgrammes, and they will be required to attend for repeat TSH measurement in 6-8 weeks to confirm that their TSH is no longer suppressed.

A mock titration will be performed in the placebo group aiming for approximately the same frequency as that likely to be required in the Levothyroxine-treated group. Wewill adopt an adaptive schedule, in which the data centre will allocate the same proportion of placebo patients to have dose adjustment (up and down) as prove to be required in the levothyroxine group. This will ensure that the burden of assessment, and number of tablets to be taken, will be the same in both the levothyroxine and placebo groups. This will also ensure that the clinical investigators will remain blind to treatment allocation.

Where a proposed up-titration of levothyroxine (or placebo) is thought to be clinically inappropriate (eg known non-adherence to IMP, recent major illness) the algorithm will be ‘over-ridden’ and the patient will not be up-titrated.

The maximum possible dose of Levothyroxine that will be prescribed is 150 micrograms. Patients will be advised to take their prescribed dose once daily in the morning before breakfast.

The daily dose of Levothyroxine used in all studies of treatment of SCH included in the Cochrane review ranged from 50-100 micrograms, with a mean dose in most studies of 50-70 micrograms /day (3). There is no good evidence that starting with a dose lower than 50 micrograms improves tolerability or reduces risk of adverse effects, and there are no short-medium term differences in changes in heart rate or blood pressure between a low dose (25 micrograms) and replacement dose (50 micrograms) strategy of initiation of Levothyroxine. Full replacement doses of Levothyroxine for overt hypothyroidism are 1.6 micrograms / Kg body weight (approximating to 100 micrograms for a 70Kg individual). While such a dose can be used right from the start, even in older subjects, we have taken a cautious approach, and have chosen 50 micrograms daily as the usual initial dose of Levothyroxine in the TRUST study, and have reduced this further to a 25 micrograms daily start dose for those with low body weight (<50Kg) or with known coronary heart disease (as evidenced by a history of previous myocardial infarction or angina pectoris).

4.3 Formulation and Source of Drug

The investigational medicinal products in this study are levothyroxine 25 and 50 micrograms tablets and matched placebo for oral use. The tablets will be white and round in shape with the tablet strength imprinted on the active and matched placebo tablets. All IMPs will be manufactured in accordance with Good Manufacturing Practice with the final Qualified Person release and distribution provided by Mawdsleys UK to study sites.

4.4 Storage and Stability

The levothyroxine/placebo tablets must be stored in the original container at room temperature below 25°C in a secure location. A shelf life of 36 months will be assigned. The study medication must only be used in accordance with the trial protocol and only for subjects enrolled into the study.

4.5 Drug Procurement

Study medication supplies will only be released to study site once all the appropriate regulatory and governance approvals are in place. The study web-portal will be used to track drug use, shipment and receipt.

4.5.1 Packing and Distribution

The medication will be supplied in blister strips (28 tablets per blister), and packaged as 4 blister strips per cardboard carton for patient distribution. All investigational medicinal products will be packaged in such a way as to maintain the study blind. All study medication will be labelled in accordance with national regulatory requirements and will have a unique pack identifier with labeling including randomisation code as supplied by the data centre. Supplies will be distributed to study centres in each country.

Detailed written information will be available to sites on study drug management including the supply of medication via post.

4.5.2 Drug Accountability

A record of study drug movements will be maintained for accountability purposes in accordance with GCP and local regulatory requirements. The records will include the quantity of investigational medicinal product dispensed to and returned from study subjects and final disposal including batch number and expiry date information.

Accountability logs will be made available for inspection by the Sponsor or their designee and Regulatory Inspectors. Detailed written information will be provided to study sites on study drug management.

4.6 Destruction of Unused Drug

Systems will be put in place for disposal of any unused study drug. Detailed written information will be provided to study sites.

4.7 Unblinding of Treatment Allocation

This will include immediate response to requests for unblinding from treating physicians or general practitioner. In this event attempt will be made to maintain blinding of the clinical research team.

4.8 Criteria for Withdrawal of Participants on Safety Grounds and Withdrawal

If overt biochemical hypothyroidism is identified (TSH ≥20 mU/L), the data-centre will require a 2nd TSH measurement with fT4 within 2 weeks; if overt biochemical hypothyroidism is confirmed (free T4 below reference range), the patient will require to stop the trial medication and attend GP for consideration of open treatment with Levothyroxine.

If biochemical hyperthyroidism (TSH<0.4 mU/L) develops in the placebo group, or occurs at 2 consecutive follow-up visits in a patient in the Levothyroxine group; i.e. persisting despite down-titration of the Levothyroxine dose the patient will require to stop the trial medication and attend GP for consideration of further assessment and treatment of hyperthyroidism.

4.9 Maintenance of trial treatment randomisation codes and procedures for unblinding.

Detailed SOPs will be developed for maintenance of trial randomisation codes and unblinding of treatment allocation.

4.10 Special warnings and precautions for use

Subclinical hyperthyroidism is associated with bone loss, increased risk of osteoporotic fractures, atrial fibrillation, heart failure and possibly worsening angina. For this reason we propose careful monitoring of thyroid function tests throughout the study, with reduction in dose of Levothyroxine in those with early biochemical evidence of over-replacement (TSH < 0.4 mU/L).

Patients with panhypopituitarism or other causes predisposing to adrenal insufficiency may react to Levothyroxine treatment, and it is advisable to start corticosteroid therapy before giving Levothyroxine to such patients. However this study effectively excludes subjects with panhypopituitarism, by requiring an elevated TSH level as a condition of entry.
Initiation or discontinuation of anti – convulsants therapy may alter levothyroxine dosage requirements
There are further special precautions relating to pregnancy, lactation and use in paediatric subjects. Such considerations are not relevant to this trial.

4.11 Interactions with other drugs

The drugs listed below may interact with the IMP given as part of the TRUST trial. However it should be noted that interactions are generally weak, and have very limited clinical relevance for the treatment of mild or subclinical hypothyroidism, with doses of Levothyroxine that avoid over-replacement (iatrogenic hyperthyroidism).

Anticoagulants

Levothyroxine increases the effect of anticoagulants and it may be necessary to reduce the anticoagulation dosage if excessive hypoprothrombinaemia and bleeding are to be avoided. However clinically relevant effects are unlikely in the context of treating subclinical hypothyroidism, as long as overtreatment with Levothyroxine is avoided.

The SOPs for the study will include detailed recommendations for warfarin monitoring for patients who are on this drug, ensuring the INR is checked soon after initiating or changing Levothyroxine dose.

Anti-convulsants

Anti-convulsants, such as carbamazepine, primidone and phenytoin, enhance the metabolism of thyroid hormones and increase requirement for thyroid hormones in hypothyroidism.

Anti-arrhythmics

Amiodarone may inhibit the de-iodination of thyroxine to tri-iodothyronine resulting in a decreased concentration of tri-iodothyronine, thereby reducing the effects of thyroid hormones. However subjects on this drug are excluded from this study.

Antidiabetics

Blood sugar levels are raised and dosage of anti-diabetic agents may require adjustment. However clinically relevant effects are unlikely in the context of treating subclinical hypothyroidism, as long as overtreatment with Levothyroxine is avoided. The SOPs for the study will include detailed recommendations for diabetes monitoring, however it is anticipated that this will not require significant changes to the diabetic monitoring that is occurring as part of routine clinical practice.

Beta Blockers

Levothyroxine (thyroxine) accelerates metabolism of propranolol. Betablockers may decrease the peripheral conversion of levothyroxine.

Antidepressant

Levothyroxine increases receptor sensitivity to catecholamines thus accelerating the response to tricyclic antidepressants (e.g. amitriptyline, imipramine).Concomitant use of tricyclic antidepressants and Levothyroxine may precipitate cardiac arrhythmias. Effects of Levothyroxine may be decreased by concomitant sertraline.

Sympathomimetics

The effects of sympathomimetic agents (e.g. adrenaline) are enhanced.

Cardiac glycosides

In theory, if Levothyroxine therapy is initiated in digitalised patients, the dose of digitalis may require adjustment. Hyperthyroid patients may need their digoxin dosage gradually increased as treatment proceeds because initially patients are relatively sensitive to digoxin. However heart rate changes in treatment of subclinical hypothyroidism are likely to be negligible, and as long as overtreatment is avoided it is expected any interaction with digoxin will not be of any clinical significance.

Antineoplastics:

Plasma concentration of Levothyroxine (thyroxine) is possibly reduced by imatinib.

Nonsteroidal anti-inflammatory drugs

False low plasma concentrations have been observed with concurrent anti-inflammatory treatment such as phenylbutazone or acetylsalicylic acid and Levothyroxine therapy.

Sex Hormones

Oestrogen, oestrogen containing product (including hormone replacement therapy) and oral contraceptives may increase the requirement of thyroid therapy dosage. Conversely, androgens and corticosteroids may decrease serum concentrations of Levothyroxine-binding globulins.

Lipid regulating drugs

Lovastatin has been reported to cause one case each of hypothyroidism and hyperthyroidism in two patients taking Levothyroxine.

General anaesthetics

Isolated reports of marked hypertension and tachycardia have been reported with concurrent ketamine administration.

Drugs affecting metabolism or absorbtion of Levothyroxine

Metabolism of Levothyroxine (thyroxine) is accelerated by rifampicin, barbituarates (these may increase dose requirements for Levothyroxine (thyroxine) in hypothyroidism).

Absorption of Levothyroxine (thyroxine) is possibly reduced by antacids, calcium salts, cimetidine, oral iron, sucralfate, colestipol, polystyrene sulphonate resin and

cholestyramine (if possible administration should be separated by 4-5 hours).

Prohibited concomitant medication

Levothyroxine; antithyroid medications (carbimazole, methimazole, propylthiouracil, potassium perchlorate); amiodarone; lithium.

5.1 METHODS OF DATA COLLECTION

Descriptive data to be recorded at screening and /or baseline

(1) Age, gender and race.
(2) Lifestyle; smoking, alcohol intake.
(3) All exclusion criteria listed in section 3.3.
(4) Known cardiovascular disease, including history of ischaemic heart disease (angina pectoris or previous myocardial infarction), cerebrovascular disease (ischaemic stroke, transient ischaemic attack) or peripheral vascular disease (intermittent claudication), or any revascularisation procedure for ischaemic vascular disease. The exact criteria for prior cardiovascular disease will be similar to those used in PROSPER (17).
(5) History of atrial fibrillation (AF).
(6) History of epilepsy.
(7) History of hypertension, diabetes mellitus or osteoporosis..
(8) Prescribed medicines and over-the counter aspirin or non-steroidal anti inflammatory drugs will be recorded at each study visit; medicine count will be used as an assessment of baseline co-morbidity.
(9) Mini-mental state examination (MMSE) score (18) will be recorded at study baseline as a descriptor of general cognitive function. However it will not be repeated or used as an outcome measure as it is insensitive to change over the time-span planned for this study.
(10) Home support services (e.g. home help, meals-on-wheels, district nursing) and home circumstances (e.g. living alone, co-habiting, standard or sheltered housing, or entry to care home), at study baseline and final review.

The follow-up time points listed above were chosen to reflect the following; at 6-8 weeks we expect most patients allocated Levothyroxine to be biochemically euthyroid, and at this time point short-term improvements (such as in thyroid-specific quality of life) will be apparent. By 1 year the medium-term effects of Levothyroxine treatment should emerge (such as on muscle function). The longer-term effects of treatment of SCH will be determined by assessment over the full course of the study.

5.2 Data Collection

Data collection will be performed by study research nurses at screening, baseline and predetermined follow-up as outlined previously. Data will be collected in a study centre or the patient's home own / place of residence.

For the screening visit; we will record exclusion criteria, results of repeat TSH and fT4, consent / decline to take part in the study and for those who have consented, their contact details. Participants in the randomised controlled trial will generate the following data:

- Baseline/ screening visit; subject characteristics, including prior cardiovascular disease, smoking, home support and Mini Mental State Examination
- Concomitant drug treatment at screening, baseline, 6-8 weeks, 12 months and annually thereafter
- Thyroid specific quality of life (ThyPRO symptom and fatigue domains) and the EuroQol5D at study baseline, 6-8 weeks and 12 months post-recruitment
  and at final review
  The ThyPRO39 will be recorded at the final study (closeout) visit. It provides an extended dataset covering a comprehensive range of thyroid-related quality of life issues, To preserve the integrity of the validated ThyPRO 39 scale we will include all domains even though some (eg goitre and eye symptoms) are not likely to be major issues in subclinical thyroid disease.
    - Memory concentration (3 items)
    - Nervousness and tension (3 items)
    - Psychological well-being (3 items)
    - Coping and mood swings (3 items)
    - Relationships with other people (3 items)
    - Daily activities (3 items)
    - Appearance (3 items)
    - Overall impact (1 item)
    - Goitre (3 items)
    - Eye Symptoms (3 items)
- The letter-digit coding test, isometric handgrip strength (best of 3) at study baseline, 12 months and at final review
- Height, weight and waist circumference will be measured at screening. Weight and waist circumference will be repeated at 12 months visit and at the final review.
- Blood pressure (systolic and diastolic) at screening, 12 months and at final review.
- Drug accountability data will be gathered for each patient including distribution date, quantity of study drug supplied, and drug supply returns including date, and quantity of tablets returned.

All sites will gather descriptive baseline and primary outcome data. Satellite sites may elect not to gather data on selected secondary outcomes (EuroQol5D, letter digit coding test, isometric handgrip) if they do not have sufficient resources to perform assessment of these secondary outcomes.

Report forms for possible cardiovascular endpoints and SAEs will be generated for the study nurses to complete; these will be entered via the trial web portal which will have an in-built notification to the Endpoints Committee. Adjudicated endpoints will also be entered via the trial web portal using separate adjudication record forms. Anonymised source documents can be uploaded by the study nurses via the trial web portal, to assist in the adjudication process, in accordance with the committee's requirements.

5.3 Data Collection Process

Data entry will be electronic, unless patient is assessed outside the study centre where data entry will be paper based with immediate transfer to electronic data entry. The Robertson Centre for Biostatistics in Glasgow will develop and manage a trial web portal, including an electronic case report form (eCRF).
The trial web portal will be in English, however, for nurse-led questionnaires and patient completed questionnaires these forms will be required in local languages. Data will either be entered in English via the eCRF or in some cases transferred to the data centre via the trial web portal.

Data validation checks will be implemented within the eCRF to give users immediate feedback on mandatory items that are missing and 'out of range' values. In addition, logic checks will be put in place to ensure no invalid data are entered. Further database validation checks will result in data queries being flagged to the sites for correction. These checks will be run routinely and will be tracked and escalated as appropriate. Any third party data validations that result in data queries will be required to be dealt with by the data source. Data will be locked at the end of the study and the lockdown procedures will be managed by the data centre. Routinely snapshots of the data will be taken in order to report to an independent data monitoring committee (IDMC) and to the authorities (annual safety reports).

All data will be securely stored for the duration of the contract and archived beyond this time for a minimum period of 5 years after study database lock. The study database will be held by RCB for the duration of the study and for a minimum period of 5 years after study close.

6.1 ASSESSMENT AND REPORTING OF ADVERSE EVENTS / SERIOUS ADVERSE EVENTS

6.2 Definitions of Adverse Events

Adverse Event (AE)

Any untoward medical occurrence in a subject to whom a medicinal product has been administered, including occurrences which are not necessarily caused by or related to that product.

Adverse Reaction (AR)

Any untoward and unintended response in a subject to an investigational medicinal product which is related to any dose administered to that subject.

6.3 Definitions of Serious Adverse Event (SAE) or Serious Adverse Reaction (SAR)

Any adverse event or adverse reaction that

a. results in death
b. is life threatening
c. requires hospitalisation or prolongation of existing hospitalisation
d. results in persistent or significant disability or incapacity
e. consists of a congenital anomaly or birth defect.
f. is otherwise considered medically significant by the investigator

i.e Important adverse events/ reactions that are not immediately life-threatening or do not result in death or hospitalisation but may jeopardise the subject or may require intervention to prevent one of the other outcomes listed in the definition above

6.4 Definition of Suspected Serious Adverse Reaction (SSAR)

Any adverse reaction that is classed in nature as serious and which is consistent with the information about the medicinal product in question set out in the summary of product characteristics (SmPC)

6.5 Definition of Suspected Unexpected Serious Adverse Reaction (SUSAR)

Any adverse reaction that is classed in nature as serious and which is not consistent with the information about the medicinal product in question set out in the summary of product characteristics (SmPC)

6.6 Recording and Reporting AEs/SAEs

We have taken particular care in devising a titration algorithm to avoid any possibility of prolonged periods of thyroid hormone over-replacement in those allocated to Levothyroxine. This should substantially reduce the risks in this group, such as of AF or cardiac failure; in epidemiological studies these problems are observed in association with biochemical hyperthyroidism and not with TSH within the reference range. Similarly for those allocated to placebo we have developed an algorithm for review that is designed to detect those who develop overt hypothyroidism who require

to start open-label Levothyroxine. These measures are designed to ensure the highest quality of patient care, including safety, of those who are randomised into the trial.

If the study demonstrates a convincing pattern of SAEs with either Levothyroxine or placebo, this would be an important endpoint in its own right. If SAEs are observed with Levothyroxine this would counterbalance any benefits observed, and would directly influence recommendations for treatment that are generated by the study.

If an association of AEs is noted with either Levothyroxine or placebo allocation this would require careful consideration as to whether it is ethical and appropriate to continue with the trial. It is necessary to ensure that any such recommendation is not influenced by the gains obtained from direct involvement in the running of the study. Therefore this is a primary remit of the IDMC, who will comprise an independent group including medical experts and an independent biostatistician.

SAEs and AEs of special interest will be recorded at all visits and telephone contacts. Patients' GPs will also be asked to report SAEs to a central office in each country. Patients will have access to a telephone hot-line (based in central office in each country) to report symptoms or concerns.

Adverse events (AEs) will be recorded, notified, assessed, reported, analysed and managed in accordance with the Medicines for Human Use (Clinical Trials) Regulations 2004 (as amended) and this protocol. Guidance is provided in Glasgow Clinical Trials Unit (GCTU) Standard Operating Procedures available at www.glasgowctu.org.

Full details of all AEs of special interest (new atrial fibrillation, heart failure, fractures, new diagnosis of osteoporosis) including the nature of the event, relationship to study drug and outcome will be recorded in the eCRF. AEs of special interest will be monitored and followed up until satisfactory resolution or stabilization.

All adverse events will be assessed for seriousness. SAEs will also be assessed for causality, expectedness and severity. This assessment will be carried out by the PI or designated medical practitioner.

Severity This should be assessed and described using the following categories:
Mild- awareness of event but easily tolerated
Moderate-discomfort enough to cause some interference with usual activity
Severe-inability to carry out usual activity.

All SAEs arising during the clinical trial will be reported to the sponsor by entering the details into the eCRF as soon as reasonably practicable and in any event within 24 hours of first becoming aware of the event. Any follow up information should also be reported.

Serious adverse events recorded in the eCRF will be transferred to the Glasgow Pharmacovigilance database.
All SUSARS will be reported to the MHRA and Ethics Committee within the following timelines:

Fatal or life threatening SUSARs: not later than 7 days after the sponsor has information that the case fulfilled the criteria for a fatal or life threatening SUSAR, and any follow up information within a further 8 days.

All other SUSARs: not later than 15 days after the sponsor has information that the case fulfilled the criteria for a SUSAR.

The GCTU Pharmacovigilance (PV) Office will report SUSARs on behalf of the CI to the MHRA via the eSUSAR reporting system and to the Ethics committee. Non-UK SUSARs will also be reported to the MRHA.

The Lead Investigator at each site will be informed about any SUSARs which have occurred during the study.

Specific regulations regarding pregnancy are not applicable to this trial. There are no risks to the foetus; male participants who take part do not need to take any contraceptive precautions who have a partner of childbearing age and female participants are beyond the childbearing age.

SAEs that occur at any time after the inclusion of the subject in the study (defined as the time when the subject signs the informed consent) up to 30 days after the subject completed or discontinued the study will be reported.

The subject is considered to have completed the study EITHER after the completion of the last visit or contact (e.g., phone contact with the investigator or designee), OR after the last dose of the study medication, whichever is later. The date of discontinuation is when a subject and/or investigator determine that the subject can no longer comply with the requirements for any further study visits or evaluations.
Stopping guidelines will be developed by the Independent Data and Safety Monitoring Committee (IDMC); it is assumed any recommendations for early stopping, such as because of overwhelming benefit for the primary outcome, will be conservative and will have no impact on the sample size calculations.

6.7 Unblinding

If the clinical investigator or attending physician deems that unblinding is necessary they will have 24-hr access to telephone unblinding through the data-centre. In the

event of a SUSAR, the sponsor (but not the investigators) will be unblinded to facilitate reporting to the MHRA.

6.8 Specific adverse events of interest

Certain potential adverse events are anticipated or likely as a result of the study and study population. The adverse events detailed below are likely to occur in the context of over replacement of Levothyroxine. Our dose titration scheme and study processes of careful monitoring of thyroid function tests are designed to ensure we avoid prolonged periods of thyroid hormone excess.
For the group allocated to placebo, there is risk of developing overt clinical hypothyroidism; again our study processes of careful monitoring of thyroid function tests are designed to avoid this scenario.

(A)
Atrial fibrillation (AF).
AF is associated with subclinical hyperthyroidism (22) and therefore is a potential risk of thyroid over-replacement for SCH. It should not occur if TSH is maintained in the normal range, however we will pay particular attention to identifying this possible adverse event.

We have developed a robust mechanism to ensure detection of atrial fibrillation. Cardiac rhythm will be determined at study baseline, and new onset AF, paroxysmal or persisting, will be diagnosed from an annual single-lead electrocardiograph, or if noted on 12-lead electrocardiograph or telemetry performed as part of hospitalisation or other clinical care, identified by inquiry about hospitalisations and out-patient visits (including for cardiac arrhythmias) at all patient contacts.

This general process of screening for atrial fibrillation has been found to be very sensitive for identifying new cases (23).
We propose to use a single-lead recorder (Omron HeartScan HCG-801-E). This provides a simple and quick assessment of cardiac rhythm; it has been shown to have high diagnostic accuracy for AF (sensitivity 99%, specificity 96%) compared to a standard 12-lead electrocardiograph (24).

(B) Heart failure.

Prevalent heart failure and incident heart failure diagnosis / hospitalisations will be recorded, as this outcome is a potential risk of thyroid hormone over-replacement.
(C) Fracture.
Musculoskeletal effects of Levothyroxine are described, including osteopenia/ osteoporosis. We will record all new fracture diagnosis and all new diagnoses of osteoporosis. Formal screening for osteoporosis is not required for this trial
.

6.9 Annual Safety Report

An annual safety report (in the format of a Development Safety Update Report) must be submitted to MHRA and REC as soon as is practicable and within 60 days of the anniversary of the issue of the Clinical Trials Authorisation. The Chief Investigator will prepare and submit this report in liaison with the PV Office.

6.10 Safety Flow Chart

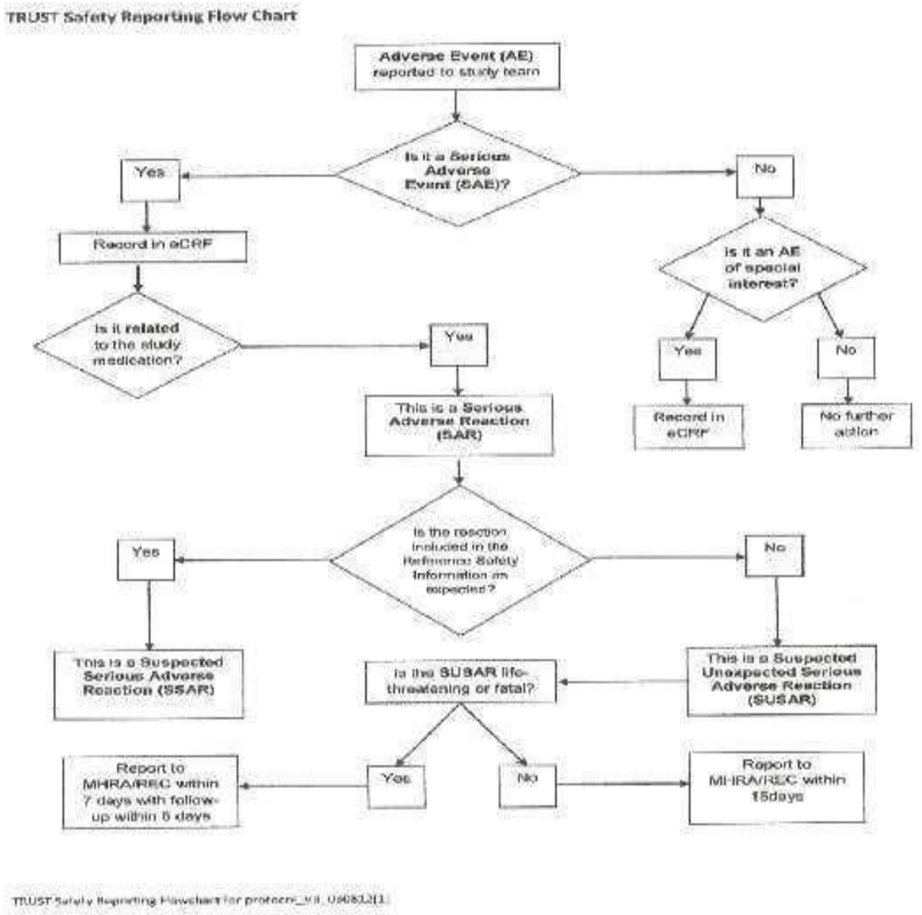

7.0 ASSESSMENT AND FOLLOW UP

The first act at the baseline study visit will be to obtain written informed consent for participation in the randomised controlled trial.

The first patient will be recruited into the study at the beginning of month 4, with recruitment taking place over 2 years. Study progress reports addressing screening numbers and recruitment are scheduled at 8, 12, 18, 24 and 28 months. The initial target number of recruits at each of these time-points, for each of the 4 Member and Associated States: is 125, 250, 375, 513 and 750 respectively. Recruit retention and adherence to treatment allocation is addressed in reports at 12, 24, 36 and 48 months. Completeness of outcome recording and cardiovascular event rates will be reported in the IDMC reports, scheduled at months 12, 24, 36 and 48.

Subjects will be reviewed face-to-face by the study nurses at recruitment, study baseline, 6-8 weeks and 12 months and annually thereafter (12, 24 and 36 month visits will be performed plus or minus 1 month) . In addition interim telephone contact will be made by study nurses at 6, 18, 30 and up to 42 months (depending on total duration of follow-up), including recording of possible cardiovascular and serious adverse events (SAEs).

Randomisation (1:1 Levothyroxine versus placebo) will be stratified by site, gender and starting dose of Levo-thyroxine and carried out using the method of randomly permuted blocks. The randomisation schedule will be prepared by the data centre (independent of the clinical investigators), implemented by the manufacturer of the matching placebo (they will package Levothyroxine and placebo), and patient allocation conducted by freephone or via the trial web portal by the study nurses.

We propose a minimum 1 year of initial follow-up, maximum 42 months (3.5 years). Subjects will be reviewed face to face by the study nurses at recruitment, study baseline, 6-8 weeks and 12 months and annually thereafter. In addition interim telephone contact will be made by study nurses at 6, 18, 30 and up to 42 months (depending on total duration of follow up), including recording of possible cardiovascular and serious adverse events (SAEs). The final review (up to 42 months) will be face-face).

Descriptive data to be recorded at visits / assessments after screening / baseline: Home support services (e.g. home help, meals-on-wheels, district nursing) and home circumstances (e.g. living alone, co-habiting, standard or sheltered housing, or entry to care home), at study baseline and final review.

Prescribed and over the counter aspirin and non-steroidal anti-inflammatory drugs will be recorded at screening, baseline, 6-8 weeks and 12 months, then annually until after review.

Disease specific quality of life will be assessed using the symptom and fatigue domains from the Thyroid-specific quality of life Patient Reported Outcome (ThyPRO) questionnaire; Of the 13 ThyPRO domains, some are not relevant for subclinical hypothyroidism (e.g. goitre, eye symptoms and cosmetic complaints), and some overlap with the general health related quality of life questions in the Euroqol; these questions therefore will be omitted from the TRUST study. The ThyPRO symptom and fatigue questionnaire will be applied at study baseline, 6-8 weeks and 12 months and at study closeout. This questionnaire will give summary scores for symptoms and for fatigue, but in addition allow for analysis of specific individual symptoms, including weight gain, depression, cold, and tiredness.

General quality of life will be assessed using the EuroQol5D, recorded at study baseline 6-8 weeks and 12 months and at final follow up. Handgrip strength will be measured using isometric dynamometry (Jamar hand dynamometer, using best of 3 attempts of dominant hand) recorded at study baseline, 12 months and final follow up. Executive cognitive function will be assessed using the letter-digit coding test recorded at study baseline and at final follow up. Blood pressure (systolic and diastolic phase V), measured at screening, 12 months and final follow up (mean of 2 measurements taken after 5 minutes sitting). Ability to perform basic activities of daily living (ADL) will be recorded using the 10 item, 20 point Barthel index, at study Baseline and final follow up. Instrumental or extended activities of daily living (IADL) will be recorded using a short (7-item) questionnaire derived from the OARS instrument. This will be recorded at study Baseline and final follow up.

The follow-up time points listed above were chosen to reflect the following; at 6-8 weeks we expect most patients allocated Levothyroxine to be biochemically euthyroid, and at this time point short-term improvements (such as in thyroid-specific quality of life) will be apparent. By 1 year the medium-term effects of Levothyroxine treatment should emerge (such as on muscle function). The long-term effects of treatment of SCH will be determined by assessment over the full course of the study, with a mean of 3 years treatment duration (maximum 42 months)

At study baseline a standard venous blood sample (40mls) will be taken for storage in the study biobank and to perform a standard full blood count. The full blood count will be repeated at 1 year and a further 8.5ml for storage in the biobank.

In addition to the above, consent will be sought at the time of screening for long term record linkage studies (using routine Scottish health data), using the information gathered to examine links of thyroid function with long term outcome in all patients who are screened for the study, and also allowing long term (post trial) outcome to be determined who those who participate in the RCT.

8.1 STATISTICAL ANALYSIS

8.2 Statistical Analysis Plan

The Robertson Centre for Biostatistics (RCB) will be responsible for writing a formal statistical analysis plan (SAP) for the trial, submitting this for review by the Steering Committee and implementing revisions. The SAP will be agreed before the final locking and unblinding of the study database.

The general strategy will be as follows: clinical outcome data will include time to first event Cox regression analysis stratified by gender in models containing the randomised treatment allocation as a covariate. Analyses will be based on the intention-to-treat principle. Tests of treatment effect will be based on the Wald test and corresponding point estimates and 95% confidence intervals for the hazard ratio for treatment will be calculated. The assumption of proportionality of hazards will be tested. Continuous variables involving measurement at follow-up and baseline will be analysed at each time point comparing treatment groups and adjusting for gender and baseline levels of the same variable using analysis of covariance (ANCOVA). In addition, such data will be analysed using repeated measures analyses (standard analyses and repeated measures regression analyses) and in terms of the final assessment for each participant.

RCB will also be responsible for creating a statistical analysis plan for the IDMC report and providing the independent IDMC statistician with pre-written and validated programmes to facilitate the provision of unblinded reports for the IDMC.

8.3 Pilot data, planned power calculations and justification of planned sample size at study commencement

A critical factor in success of any randomised controlled trial is feasibility of recruitment. Pilot work on laboratory databases was conducted in Glasgow and in Leiden. In the Glasgow clinical laboratory in 2010 (1 year) there were 8,866 TSH results in the range of interest (≥4.6, ≤19.9mU/L) from patients > 65yrs. Of these 1,684 can be excluded as known to be on Levothyroxine, leaving 7,182 who might be eligible for our trial; of this group 70.2% were female, 17.4% were ≥ 85 years, and 16.1% had a TSH of ≥10mU/L. However some of these subjects will not be eligible for our study, due to free thyroxine levels not in the reference range or other exclusion factors; as a conservative estimate we might anticipate only 50% (3,591) would be eligible and willing for repeat thyroid function tests in our screening process. Of these, thyroid function tests might be expected to have normalised in around one-third on repeat testing at the screening visit, leaving around 2,400 patients who could be asked consent for randomisation into the study. We have proposed 2 years for recruitment of 750 subjects per site. Therefore to recruit half this number in the first year of recruitment in Glasgow we would need to consent and randomise 375 / 2,400 or 15.6% of patients who are eligible after screening. In practice we hope to

recruit around 20% of subjects who have thyroid function tests done at the screening visit.

Pilot data were available from a single laboratory in Leiden; in 2010, 24,541 patients 65 years or older had thyroid function checked, with 1,352 (5.5%) showing TSH of (≥4.6, ≤19.9mU/L. Therefore to give a similar size screening population to Glasgow around 5-6 such laboratories in the Netherlands would be required. We anticipated similar numbers of laboratories will be used to generate the screening populations in Switzerland and Ireland.

Outcomes and outcome recording: we will aim for data to be gathered for over 95% of patients followed up for cardiovascular events and deaths, and over 90% for all other outcomes (including thyroid specific and general health-related quality of life, muscle strength and cognition). We are aiming to record 565 cardiovascular events over 9,000 years of patient follow-up. If 3,000 patients are followed up for an average of 36 months, they will have on average 4 on-treatment measures of thyroid function i.e. 12,000 TSH measurements during follow-up.

Adherence to treatment allocation: drop-ins (where subjects allocated to placebo are prescribed Levothyroxine) and drop outs (where subjects allocated to Levothyroxine stop this treatment) are each estimated at less than 5% at 1 year and less than 10% at the end of the study (mean 3 years follow-up).

The first patient will be recruited into the study at the beginning of month 4, with recruitment taking place over 2 years. Study progress reports addressing screening numbers and recruitment are scheduled at 8, 12, 18, 24 and 28 months. The target number of recruits at each of these time-points, for each of the 4 Member and Associated States, is 125, 250, 375, 513 and 750 in total. Recruit retention and adherence to treatment allocation is addressed in reports at 12, 24, 36 and 48 months. Completeness of outcome recording and cardiovascular event rates will be reported in the IDMC reports, scheduled at months 12, 24, 36 and 48.

8.4 Revised power calculations and sample size

Due to significantly lower than expected recruitment rates at all study sites, it is expected that substantially fewer subjects will be recruited into the study. See addendum p57 for revised recruitment numbers and discussion on revised primary and secondary end-points.

8.5 Statistical Methods

Clinical outcome data will include time to first event Cox regression analysis stratified by gender in models containing the randomised treatment allocation as a covariate. Analyses will be based on the intention-to-treat principle. Tests of treatment effect will be based on the Wald test and corresponding point estimates and 95% confidence

intervals for the hazard ratio for treatment will be calculated. The assumption of proportionality of hazards will be tested.

Continuous variables involving measurement at follow-up and baseline will be analysed at each time point comparing treatment groups and adjusting for gender and baseline levels of the same variable using analysis of covariance (ANCOVA). In addition, such data will be analysed using repeated measures analyses (standard analyses and repeated measures regression analyses) and in terms of the final assessment for each participant. For disease-specific and general quality of life, greatest effect will be expected after 1 year of treatment, and for these endpoints this will be the primary time-point for analysis.

A detailed review of power calculations for reduced recruitment numbers is provided in an addendum p57.

Randomisation (1:1 Levothyroxine versus placebo) will be stratified by site, gender and starting dose of levothyroxine and carried out using the method of randomly permuted blocks.

9 END OF TRIAL

The subject is considered to have completed the study EITHER after the completion of the last visit or contact (e.g., phone contact with the investigator or designee), OR after the last dose of the study medication, whichever is later. The date of discontinuation is when a subject and/or investigator determine that the subject can no longer comply with the requirements for any further study visits or evaluations.

For the purposes of regulatory requirements the end of the trial is defined as the date of the last investigational visit for the last patient undergoing protocol treatment.

Patients who either complete or withdraw from study treatment will be referred back to their General Practitioner for their on-going care. Any future treatment would be at the discretion of the patient's GP with costs being borne by the NHS as part of routine patient care. We aim to inform the participant (if they wish) and their general practitioner (GP) which arm of the study they had been allocated to i.e. placebo or levothyroxine within 15 working days of completing a participant’s final study visit. This will be done by letter. This information should aid discussions between the participant and their GP regarding any further treatment.

Only one member of the study team will be “unblinded” in the course of releasing this information and this individual will not be involved in a gathering study data or assessing serious adverse events or possible Endpoints.

10 TRIAL MANAGEMENT

The investigators institution(s) will permit trial related monitoring and audits, ethical reviews and regulatory inspections by providing direct access source data/documents.

10.1 Study administrative team

A study administrative office will be sited in Glasgow. The study will be guided by a central steering committee, which will include external expert advisors, patient advocacy (Thyroid Federation International), independent ethics advisor and representatives from all consortium partners. Potential cardiovascular endpoints will be reviewed and adjudicated by an endpoints committee. An Independent Data and Safety Monitoring Committee (IDMC) will be established, to review outcome and SAE data and advise the steering committee and sponsors on continuation of the study. Each of the 4 Member / Associated States will establish a local organising committee to deal with operational issues.

10.2 Trial Steering Committee

The Trial Steering Committee is the ultimate scientific decision-making body for the study. It is chaired by the Coordinator (or their Deputy), and will meet regularly throughout the lifespan of the project.

Membership will also include study biostatistician (Prof Ford), and leads for each other (non-UGLA) beneficiary (UCC – Dr Kearney, LUMC – Prof Gussekloo, UBERN - Dr Rodondi, LAVA – Prof Westendorp). External experts will include Dr Doug Bauer, from the US team who are proposing a complimentary NIH grant application. The leading international patient representative group for thyroid dysfunction and disease, Thyroid Federation International, will be represented by their president, Yvonne Anderson (or deputy). To be quorate, each beneficiary organisation will be represented by at least one person (from the named collaborators), however all named collaborators from each of the 5 main participants will be eligible to attend.

The Steering Committee will have a formal charter. Responsibilities include:

- Providing overall project management policy.
- Making formal decisions on the project and project strategy (including proposing to the EU any changes to the work plan and / or the budget).
- Ensuring effective dissemination and knowledge management, including IPR and determining the publication strategy.

10.3 Independent Data and Safety Monitoring Committee (IDMC)

An IDMC will be established to include a minimum of two independent medical experts (covering the domains of geriatric medicine, thyroid and cardiovascular disease; one of the academic clinicians will act as chair) and an independent biostatistician. The identities of the independent IDMC members have yet to be confirmed; the director of the Robertson Centre for Biostatistics (Professor Ian Ford) will liaise with the committee, attend open sessions, and ensure that the committee is provided with adequate information about study progress and results.

The IDMC will have a formal charter; this will outline the responsibilities of the IDMC members, Data Centre and the co-sponsors. Responsibilities include:

- To protect the safety of patients recruited to the trial.
- Advising Steering Committee, Sponsors and EU if it is safe and appropriate to continue with the study.
- Scrutinising recruitment and endpoint rates, and providing reports for the Project Office to forward to the Steering and ethical committees, regulatory bodies and for the EU.
- Examining information provided by the Data Centre on study recruitment, adverse events and outcomes and providing reports for the Project Office to forward to the Steering Committee, ethics committees, regulatory bodies, study sponsors, and the EU.

10.4 National organising committees

Each participating Member / Associated state will establish a National organising committee, chaired by one of the main study applicants. National organising committee responsibilities will include:

- Obtaining national ethical committee approval including for protocol amendments.
- Establishing local standard operating procedures for screening, recruitment and follow-up of randomised patients (including endpoints).
- Pharmacovigilance reporting to appropriate regulatory authorities within each Member / Associated State.
- Preparing language-specific website content, record forms (in consultation with the Data Centre), patient information sheets, consent forms, posters and newsletters.
- Organising National launch and the National study closing meetings.
- Providing information for Project Office on staff recruitment and budgetary management.
- Ensuring prompt information transfer to the Data Centre, including patient screening and recruitment, results of in-study TSH from the regional clinical laboratories, and follow-up including all study endpoints.
- To inform the Project Office of any significant problems in the conduct of the study.

11 CO-SPONSOR RESPONSIBILITIES (NHS GREATER GLASGOW AND CLYDE/UNIVERSITY OF GLASGOW)

Prior to study initiation, a non-commercially funded clinical trial co-sponsorship agreement will be put in place between NHS Greater Glasgow and Clyde and University of Glasgow. The role and liabilities each organisation will take under Clinical Trials Regulations, 2004 (SI 2004:1031) are laid out in this agreement signed by both organisations. The University of Glasgow shall be responsible for carrying out the obligations and responsibilities set out in the aforementioned agreement, and shall be deemed "sponsor" for the purposes of Part 3 of the regulations in relation to the study. NHS Greater Glasgow and Clyde shall be responsible for carrying out the obligations and responsibilities set out in the agreement, and shall be deemed "sponsor" for the purposes of Parts 4, 5, 6 and 7 of the regulations in relation to the study.

Each participating member/associated state will designate a legal entity to operate as sponsor. The UK co-sponsors will delegate sponsor responsibilities to each identified legal entity and this will be defined in a 'Research Agreement for the performance of an intergroup clinical trial'. A fully executed agreement will be implemented with each participating member/associated state prior to the TRUST study starting in that country.

11.1 Study monitoring and auditing

Study monitoring visits will be conducted according to a study-specific monitoring plan devised by NHS Greater Glasgow and Clyde and subsequent monitoring reports will be reviewed by NHS Greater Glasgow and Clyde. The trial will undergo a Monitoring Risk assessment; this assessment will identify the level of monitoring and audit required. At a minimum, each site will be monitored before the study commences (Study Initiation), study visit(s) and at the end of the trial (Study Close Out Visit). Additional monitoring visits may be undertaken if required and the study may be subject to routine or for-cause audit visits. Investigators and site staff will be notified in advance of any audit and/or monitoring visits.

Sponsors outwith the UK will identify the level of monitoring required and develop a monitoring plan, the UK monitoring plan will be provided on request.

11.2 Protocol amendments

Any change in the study protocol will require an amendment. Any proposed protocol amendments will be initiated by the CI and any required amendment forms will be submitted to the MHRA, REC (committee which provided original ethical opinion) and sponsor. The CI will determine whether an amendment is non-substantial or substantial on behalf of the co-sponsors. All amended versions of the protocol will be signed by the CI and co-sponsor's representative. Before the amended protocol can be implemented (or sent to other participating sites) favourable opinion/approval will be

sought from the original reviewing REC, MHRA and site Research and Development office.

Each participating member/associated state must follow their own sponsor process which falls in line with each country's governing laws.

12 INDEMNITY AND INSURANCE

The TRUST trial is co-sponsored by NHS Greater Glasgow and Clyde and University of Glasgow. The co-sponsors will be liable for negligent harm caused by the design of the trial. NHS Indemnity is provided under the Clinical Negligence and Other Risks Indemnity Scheme (CNORIS). As the substantive employer of the CI and as co-sponsor of the trial, the University of Glasgow also has insurance with Royal and Sun Alliance. It has been confirmed prior to the trial starting that insurance cover will be provided automatically under the current policy. The insurance cover will be subject to NHS indemnity being in place and REC approval being obtained.

The NHS has a duty of care to patients treated, whether or not the patient is taking part in a clinical trial, and the NHS remains liable for clinical negligence and other negligent harm to patients under this duty of care. There are no specific arrangements for no-fault compensation.

The indemnity and insurance statement will only apply to UK participating site(s). Each participating member/associated state must ensure that appropriate insurance is in place prior to the TRUST study starting.

13 FUNDING

Funding for the study is provided through the European seventh framework programme FP7.
The total study costs (just under €6M) equate to approximately €2,000 per patient recruited, which is a generally realistic and appropriate sum for a long-term study with multiple clinical endpoints.

14 PUBLICATION

The study is registered with the clinical trials database clinicaltrials.gov (NCT01660126)..

We are in a strong position to ensure effective dissemination of the results of the TRUST study, including early adoption into clinical practice. The Leyden Academy on Vitality and Ageing (LAVA) is well placed to play a coordinating role in this activity, given its role as a knowledge centre with an education and research program in the field of ageing, vitality and geriatric medicine.

The patient advocacy group Thyroid Federation International will play a key role in planning the dissemination strategy and approving outputs to ensure methods and content fit with the public need.

The study team has an impressive record of publication of their research in high-quality peer-reviewed journals, and this will form a key part of the primary dissemination strategy.

The Institute for Evidence-based Medicine in Old Age (the Netherlands) is ideally placed to ensure that the results of the study are considered by, and included in the leading clinical guidelines. This will be facilitated by the inclusion of study data in high-quality systematic reviews; using links through the Cochrane Field for Older People results of TRUST will be offered for the update of the Cochrane systematic review of treatment of subclinical hypothyroidism, allowing for independent scientific interpretation. We will also request of the European Thyroid Association Executive Committee that they endorse this trial, and consequently, use the outcomes to inform clinical advisory / guideline statements.

Scientific publications will be targeted for high ranking peer-reviewed journals; publication will be open access manner where possible. Scientific publications will include:

- The TRUST rationale and design. This will be published in the first phases of the study to enhance visibility of the trial for researchers around the world and to ensure full transparency.
- A review of the current state of the art regarding the evidence for treatment of subclinical hypothyroidism in old age.
- The main study results will be published in a high ranking peer-reviewed journal reporting the primary study results and interpretation.
- The steering committee will actively search for collaborations with other trials (such as the USA NIH initiative) to jointly analyse results and publish on consensus regarding implications for treatment of subclinical hypothyroidism in old people around the globe.

Scientific presentations

In parallel to the scientific publication, in first instance the rational, design and progress of the TRUST will be presented in leading conferences in various domains (cardiology, endocrinology, geriatrics). A standard presentation and slide-set in multiple languages will facilitate presentation of this information by all participating researchers.

Inclusion of study results in high quality systematic reviews and clinical guidelines

In cooperation with the Cochrane collaboration the results of TRUST will be offered for the update of the Cochrane systematic review of treatment of subclinical hypothyroidism, allowing for independent scientific interpretation, placing results in context and maximising understanding of the implication of the trial.

In cooperation with the Institute for Evidence-based Medicine in Old Age (the Netherlands) a strategy will be employed to disseminate the results of the trial in clinical guidelines.

We will also request of the European Thyroid Association Executive Committee that they endorse this trial, and consequently, use the outcomes to inform clinical advisory / guideline statements.

Neither the complete nor any part of the results of the study carried out under this protocol, nor any of the information provided by the Co-Sponsor for the purposes of performing the study, will be published or passed on to any third party without the consent of the study Co-Sponsor. Any investigator involved with this study is obligated to provide the Co-Sponsor with complete test results and all data derived from the study.

15 ARCHIVING

Archiving of clinical trial documents will be performed following Glasgow Clinical Trials Unit SOPs. Each participating member/associated state will follow their sponsor process for archiving.

16
REFERENCES

(1) Jones DD, May KE, Geraci SA. Subclinical thyroid disease. Am J Med 2010 June;123(6):502-4.

(2) Rodondi N, den Elzen WP, Bauer DC, Cappola AR, Razvi S, Walsh JP et al. Subclinical hypothyroidism and the risk of coronary heart disease and mortality. JAMA 2010;304(12):1365-74.

(3) Villar HC, Saconato H, Valente O, Atallah AN. Thyroid hormone replacement for subclinical hypothyroidism. Cochrane Database of Systematic Reviews (3):CD003419, 2007 2007;(3):CD003419.

(4) Hollowell JG, Staehling NW, Flanders WD, Hannon WH, Gunter EW, Spencer CA et al. Serum TSH, T(4), and thyroid antibodies in the United States population (1988 to 1994): National Health and Nutrition Examination Survey (NHANES III). Journal of Clinical Endocrinology & Metabolism 2002;87(2):486-8.

(5) Razvi S, Ingoe L, Keeka G, Oates C, McMillan C, Weaver JU. The beneficial effect of L-thyroxine on cardiovascular risk factors, endothelial function, and quality of life in subclinical hypothyroidism: randomized, crossover trial. Journal of Clinical Endocrinology & Metabolism 2007 May;92(5):1715-23.

(6) Gussekloo J, van EE, de Craen AJ, Meinders AE, Frolich M, Westendorp RG. Thyroid status, disability and cognitive function, and survival in old age. JAMA 2004 December 1;292(21):2591-9.

(7) McMillan C, Bradley C, Razvi S, Weaver J. Evaluation of new measures of the impact of hypothyroidism on quality of life and symptoms: the ThyDQoL and ThySRQ. Value Health 2008 March;11(2):285-94.

(8) Vigario P, Teixeira P, Reuters V, Almeida C, Maia M, Silva M et al. Perceived health status of women with overt and subclinical hypothyroidism. Medical Principles & Practice 2009;18(4):317-22.

(9) Reuters VS, Teixeira PF, Vigario PS, Almeida CP, Buescu A, Ferreira MM et al. Functional capacity and muscular abnormalities in subclinical hypothyroidism. American Journal of the Medical Sciences 2009 October;338(4):259-63.

(10) Valenti G, Fabbo A. Subclinical hypothyroidism in the elderly. Archives of Gerontology & Geriatrics 1996;22:Suppl-92.

(11) Razvi S, Weaver JU, Pearce SH. Subclinical thyroid disorders: significance and clinical impact. Journal of Clinical Pathology 2010 May;63(5):379-86.

(12) Mainenti MR, Vigario PS, Teixeira PF, Maia MD, Oliveira FP, Vaisman M. Effect of levothyroxine replacement on exercise performance in subclinical hypothyroidism. Journal of Endocrinological Investigation 2009 May;32(5):470-3.

(13) Rodondi N, Newman AB, Vittinghoff E, Rekeneiri N, Satterfield S, Harris TB et al. Subclinical hypothyroidism and the risk of heart failure, other cardiovascular events and death. Arch Intern Med 2005;165:2460-6.

(14) Simonsick EM, Newman AB, Ferrucci L, Satterfield S, Harris TB, Rodondi N et al. Subclinical hypothyroidism and functional mobility in older adults. Archives of Internal Medicine 2009 November 23;169(21):2011-7.

(15) Surks MI, Ortiz E, Daniels GH, Sawin CT, Col NF, Cobin RH et al. Subclinical thyroid disease: scientific review and guidelines for diagnosis and management. JAMA 2004 January 14;291(2):228-38.

(16) Helfand M, U.S.Preventive Services Task Force. Screening for subclinical thyroid dysfunction in nonpregnant adults: a summary of the evidence for the U.S. Preventive Services Task Force. Annals of Internal Medicine 2004 January 20;140(2):128-41.

(17) Shepherd J, Blauw G.J., Murphy M.B., Cobbe SM, Bollen ELEM, Buckley BM et al. A prospective study of Pravastatin in the Elderly at Risk (PROSPER): The Design. Am J Cardiol 1999;84:1192-7.

(18) Folstein MF, Folstein SE, McHugh PR. "Mini-Mental State": a practical method for grading the cognitive state of patients for the clinician. J Psychiatr Res 1975;12:189-98.

(19) Ford I, Blauw GJ, Murphy MB, Shepherd J, Cobbe SM, Bollen ELEM et al. A prospective study of Pravastatin in the elderly at risk (PROSPER): screening experience and baseline characteristics. Current Controlled Trials in Cardiovascular Medicine 2002;3:8.

(20) Shepherd J, Blauw G.J., Murphy M.B., Bollen ELEM, Buckley BM, Cobbe SM et al. Pravastatin in elderly individuals at risk of vascular disease (PROSPER): a randomised controlled trial. Lancet 2002;360:1623-30.

(21) Antithrombotic Trialists' (ATT) Collaboration. Aspirin in the primary and secondary prevention of vascular disease: collaborative meta-analysis of individual

participant data from randomised trials. Lancet 2009;373(9678):1849-60.

(22) Cappola AR, Fried LP, Arnold AM, Danese MD, Kuller LH, Burke GL et al. Thyroid status, cardiovascular risk, and mortality in older adults. JAMA 2006 March 1;295(9):1033-41.

(23) Mozaffarian D, Psaty BM, Rimm EB, Lemaitre RN, Burke GL, Lyles MF et al. Fish intake and risk of incident atrial fibrillation. Circulation 2004 July 27;110(4):368- 73.

(24) Kalesche G, Hoffmann B, Drewitz I, Steinbeck G, Naebauer M, Goette A et al. Prospective, multicentre validation of a simple, patient-operated electrocardiographic system for the detection of arrythmias and electrocardiographic changes. Europace 2009;11:1362-8.

(25) Shepherd J, Blauw G.J., Murphy M.B., Bollen ELEM, Buckley BM, Cobbe SM et al. Pravastatin in elderly individuals at risk of vascular disease (PROSPER): a randomised controlled trial. Lancet 2002;360:1623-30.

(26) Bremner AP, Feddema P, Joske DJ, Leedman PJ, O'Leary PC, Olynyk JK, Walsh JP. Significant association between thyroid hormones and erythrocyte indices in euthyroid subjects. Clinical Endocrinology 2012; 76(2):304-311.

APPENDICES

- Declaration of Helsinki ☐
- Copies of all Questionnaires, outcomes assessments
- Validation of specific tests being used (hand dynamometer) ☐
- SmPC of thyroxine ☐
- CRF Template ☐
- Patient Diaries ☐
- Letter of invitation sent to subjects ☐
- Patient Information Leaflet (PIL) and Informed Consent ☐
- Letter to GP ☐
- 24 hour emergency cover procedures ☐
- Data Monitoring Committee contacts ☐

Appendix 1

Outcomes scales – application and scoring rules

a) Mini-mental State Examination
b) The symptom and fatigue domains from the Thyroid-specific Quality of Life patient- reported outcome measure (ThyPRO)
c) EuroQOL-5D
d) Hand Grip Strength
e) Letter Digit Coding Test (LDCT)
f) The Barthel Index of Activities of Daily Living
g) Older Americans Resources and Services (OARS) 7-item instrument

Folstein Mini Mental State Examination

Overview

The mini-mental state examination (MMSE) is a popular screening test for cognitive impairment and is standard in many health-care systems. Using direct questioning, 8 different cognitive domains are tested across 11 items - with a total sum-score of 0-30. A total core of 24 or less is usually taken to represent dementia.

**Task**	**Instructions**	**Scoring**	
Date Orientation	"Tell me the date?" Ask for omitted items.	One point each for year, season, date, day of week, and month	5
Place Orientation	"Where are you?" Ask for omitted items.	One point each for state, county, town, building, and floor or room	5
Register 3 Objects	Name three objects slowly and clearly. Ask the patient to repeat them.	One point for each item correctly repeated	3
Serial Sevens	Ask the patient to count backwards from 100 by 7.Stop after five answers. (Or ask them to spell "world" backwards.)	One point for each correct answer (or letter)	5
Recall 3 Objects	Ask the patient to recall the objects mentioned above.	One point for each item correctly remembered	3
Naming	Point to your watch and ask the patient "what is this?" Repeat with a pencil.	One point for each correct answer	2

Repeating a Phrase	Ask the patient to say "no ifs, ands, or buts."	One point if successful on first try	1
Verbal	Give the patient a plain	One point for each correct action	3

Commands	piece of paper and say "Take this paper in your right hand, fold it in half, and put it on the floor."		
Written Commands	Show the patient a piece of paper with "CLOSE YOUR EYES" printed on it.	One point if the patient's eyes close	1
Writing	Ask the patient to write a sentence.	One point if sentence has a subject, a verb, and makes sense	1
Drawing 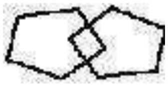	Ask the patient to copy a pair of intersecting pentagons onto a piece of paper.	One point if the figure has ten corners and two intersecting lines	1
	A score of 24 or above is considered normal		30
Scoring			

Folstein MF, Folstein SE, McHugh PR. "Mini-Mental State": a practical method for grading the cognitive state of patients for the clinician. J Psychiatr Res 1975;12:189-98.

The Thyroid-specific Quality of Life patient-reported outcome measure (ThyPRO).

Overview

The thyroid-specific quality of life (QoL) patient-reported outcome (PRO) measure for benign thyroid disorders has strong clinical validity and good test–retest reliability. The full scale consists of 84 items (plus a general quality of life question) and takes on average 14 minutes to complete.

Of the 13 ThyPRO domains, some are not relevant for subclinical hypothyroidism (e.g. goitre [11 questions], eye symptoms [8 questions] and cosmetic complaints [6 questions]) and other questions overlap with the general health related quality of life questions in the Euroqol; we have omitted these questions from the primary outcomes in the TRUST study.

We will ask the ThyPro questions for 2 domains (symptoms, fatigue / vitality), adding up to 19 questions.
In addition, at study close out only, we will ask an additional 28 questions to allow calculation of the ThyPRO39 as a secondary outcome measure. This comprehensive assessment has 11 domains, including 8 that are additional to our primary ThyPRO assessments. These domains are:

1. Memory concentration (3 items)
2. Nervousness and tension (3 items)
3. Psychological well-being (3 items)
4. Coping and mood swings (3 items)
5. Relationships with other people (3 items)
6. Daily activities (3 items)
7. Appearance (3 items)
8. Overall impact (1 items)
9 Goitre (3 items)
10 Eye Symptoms (3 items)

Validity and reliability of the novel thyroid-specific quality of life questionnaire, ThyPRO. Watt T., Hegedus L., Groenvold M., Bjorner J.B., Rasmussen A.K., Bonnema S.J., Feldt-Rasmussen U. Journal of Endocrinology, Supplement. 162 (1) (pp 161-167), 2010.

Development of a Short Version of the Thyroid-Related Patient-Reported Outcome ThyPRO. Watt T, Bjorner JB, Groenvold M, Cramon P, Winther KH, Hegedus L, Bonnema SJ, Rasmussen K, Ware Jr JE, Feldt-Rasmussen U. Thyroid, 25 (10)(pp1069-1079), 2015.

Quality of Life Questionnaire for Patients with Thyroid Disease

Please base your answers on how you have been feeling in general during the past 4 weeks.

1 The first questions are about symptoms.

During the past four weeks have you:

	Not at all	A little	Some	Quite a bit	Very much
1a- had trembling hands?	□	□	□	□	□
1b - had a tendency to sweat a lot?	□	□	□	□	□
1c - experienced palpitations (rapid heart beat)?	□	□	□	□	□
1d - experienced shortness of breath?	□	□	□	□	□
1e - been sensitive to heat?	□	□	□	□	□
1f - been sensitive to cold?	□	□	□	□	□
1g - had an increased appetite?	□	□	□	□	□
1h - had loose stools?	□	□	□	□	□
1i- had an upset stomach?	□	□	□	□	□
1j - had swollen hands or feet?	□	□	□	□	□
1k - had dry skin?	□	□	□	□	□
1l - had itchy skin?	□	□	□	□	□

2. The following questions are about fatigue

During the past four weeks have you:

	Not at all	A little	Some	Quite a bit	Very much
2a been tired?	□	□	□	□	□
2b been exhausted?	□	□	□	□	□
2c had difficulty getting motivated to do anything at all?	□	□	□	□	□
2d felt worn out?	□	□	□	□	□

3. The following questions are about vitality

During the past four weeks have you:

	Not at all	A little	Some	Quite a bit	Very much
3a felt full of life?	□	□	□	□	□
3b felt energetic?	□	□	□	□	□
3c been able to cope with the demands of your life?	□	□	□	□	□

Scale content of the ThyPRO-39

The twelve ThyPRO-39 scales consist of the following items, summarized within each scale to form a scale score for each scale ranging 0-100. All question responses are graded as Not at all, A little, Some, Quite a bit, Very much.

Eleven questions (numbers 4, 5, 6, 7, 8, 9, 10, 11, 15, 16, 17) that have already been included in the assessment of hyperthyroid or hyperthyroid symptoms or tiredness will not be repeated. Therefore we will ask an additional 28 questions to complete the ThyPRO-39.

Goitre symptoms:

During the past 4 weeks have you:

1) had the sensation of fullness in the neck?

2) felt pressure in your throat?

3) felt discomfort swallowing?

Hyperthyroid symptoms:

During the past 4 weeks have you:

4) had trembling hands?

5) had a tendency to sweat a lot?

6) experienced palpitations (rapid heart beat)?

7) had an upset stomach?

Hypothyroid symptoms:

During the past 4 weeks have you:

8) been sensitive to cold?

9) had swollen hands or feet?

10) had dry skin?

11) had itchy skin?

Eye symptoms:

During the past 4 weeks have you:

12) had the sensation of dryness or “grittiness” in the eyes?

13) had impaired vision?

14) been very sensitive to light?

Tiredness:

During the past 4 weeks have you:

15) been tired?

16) had difficulty getting motivated to do anything at all?

17) felt energetic?*

Cognitive problems:

During the past 4 weeks have you:

18) had difficulty remembering?

19) had slow or unclear thinking?

20) had difficulty concentrating?

Anxiety:

During the past 4 weeks have you:

21) felt afraid or anxious?

22) felt tense?

23) felt uneasy?

Depressivity:

During the past 4 weeks have you:

24) felt sad?

25) felt unhappy?

26) had self-confidence?*

Emotional Susceptibility:

During the past 4 weeks have you:

27) noticed you easily felt stressed?

28) had mood swings?

29) felt in control of your life?*

Impaired Social life:

During the past 4 weeks has your thyroid disease caused you to:

30) have difficulty being together with other people (for example, spouse, children, boy/girlfriend, friends, or others)?

31) feel you were a burden to other people?

32) have conflicts with other people?

Impaired Daily life:

During the past 4 weeks has your thyroid disease caused you to:

33) have difficulty managing your daily life?

34) not be able to participate in life around you?

35) feel as if everything takes longer to do?

Cosmetic Complaints:

During the past 4 weeks have you:

36) has your thyroid disease affected your appearance (for example, swelling of the neck, eye changes, weight changes)?

37) have you been bothered by other people looking at you?

38) has your thyroid disease influenced which clothes you wear?

In addition, ThyPRO contains one item not included in any multi-item scale:

During the past 4 weeks

39) has your thyroid disease had a negative effect on your quality of life?

*Positively worded items are scored reversely when constructing scales

The EuroQol5D

Overview

EQ-5D™ is a standardised instrument for use as a measure of health outcome. Applicable to a wide range of health conditions and treatments, it provides a simple descriptive profile and a single index value for health status.

EQ-5D is designed for self-completion by respondents and is ideally suited for use in postal surveys, in clinics and face-to-face interviews. It is cognitively simple, taking only a few minutes to complete. Instructions to respondents are included in the questionnaire.

The EQ-5D self-report questionnaire (EQ-5D) essentially consists of two pages comprising the EQ-5D descriptive system and the EQ Visual Analogue Scale. The respondent is asked to indicate his/her health state by ticking (or placing a cross) in the box against the most appropriate statement in each of the 5 dimensions. This decision results in a one-digit number expressing the level selected for that dimension. The digits for five dimensions can be combined in a five-digit number describing the respondent's health state.

Adapted from: *EQ-5D homepage http://www.euroqol.org/ (last accessed July 2011).*

EuroQol--a new facility for the measurement of health-related quality of life. The EuroQol Group. Health Policy 1990 December;16(3):199-208.

The EuroQol5D

Mobility

I have no problems in walking about □
I have some problems in walking about □ I
am confined to bed □

Self-care

I have no problems with self-care □ I
have some problems washing or dressing myself □ I
am unable to wash or dress myself □

Usual activities (e.g. work, study, housework, family or leisure activities)

I have no problems with performing my usual activities □
I have some problems with performing my usual activities □
I am unable to perform my usual activities □

Pain/ discomfort

I have no pain or discomfort □ I
have moderate pain or discomfort □ I
have extreme pain or discomfort □

Anxiety/ Depression

I am not anxious or depressed □ I
am moderately anxious or depressed □ I
am extremely anxious or depressed □

Hand Grip Strength

Will be measured using isometric dynamometry.
A Jamar hand dynamometer will be used, recorded score will be best of 3 attempts using dominant hand.

Fried LP, Tangen CM, Walston J, Newman AB, Hirsch C, Gottdiener J et al. Frailty in older adults: evidence for a phenotype. Journals of Gerontology Series A-Biological Sciences & Medical Sciences 2001 March;56(3):M146-M156.

Letter Digit Coding Test (LDCT)

The letter digit coding test is used to measure the speed of processing of general information and draws upon several cognitive processes simultaneously, such as visual scanning, perception, visual memory, visuoconstruction and motor functions. The subject is given a piece of paper with nine letters corresponding with nine digits. Next on this piece of paper are three rows of digits with empty spaces below them. The subject is asked to fill in as many corresponding letters as possible in 90 seconds

Smith A. The Symbol Digit Modalities Test. A neuropsychological test for economic screening of learning and other cerebral disorders. Learning Disorders 1968;3:82-91.

The Barthel Index of Activities of Daily Living

Overview

The Barthel Index (BI) is an ordinal scale describing basic (or personal) activities of daily living (ADL).

First used around 1955, Barthel's eponymous scale quickly became popular in rehabilitation, such that it is now arguably the most popular ADL scale in clinical practice.

The scale describes ten tasks and is scored according to amount of time or assistance required by the patient. Total score is from 0-100, with lower scores representing greater nursing dependency.

Several authors have proposed modifications to Barthel's original scale. Distinguishing between these BI scales is crucial, as even minor changes to scales can produce substantial differences in scoring. It is unfortunate that many of these BI variations maintain the descriptor "Barthel Index". There is no consensus on the optimal version. For this study we will use the 10 item scale, scoring 0-20 as described by Collin and Wade. In additional to recording bladder function we will note specifically whether or not the patient has a urinary catheter.

Adapted from: *Quinn TJ, Langhorne P, Stott DJ. Barthel Index for stroke trials– development, properties and application. Stroke 2011; 42:1146-1151.*

The Barthel Index of Activities of Daily Living

FEEDING

0 = unable

1 = needs help cutting, spreading butter, etc., or requires modified diet

2 = independent

BATHING

0 = dependent

1 = independent (or in shower)

GROOMING

0 = needs to help with personal care

1 = independent face/hair/teeth/shaving (implements provided)

DRESSING

0 = dependent

1 = needs help but can do about half unaided

2 = independent (including buttons, zips, laces, etc.)

BOWELS

0 = incontinent (or needs to be given enemas)

1 = occasional accident

2 = continent

BLADDER

0 = incontinent, or catheterized and unable to manage alone

1 = occasional accident

2 = continent

TOILET USE

0 = dependent

1 = needs some help, but can do something alone

2 = independent (on and off, dressing, wiping)

TRANSFERS (BED TO CHAIR AND BACK)

0 = unable, no sitting balance

1 = major help (one or two people, physical), can sit

2 = minor help (verbal or physical)

3 = independent

MOBILITY (ON LEVEL SURFACES)

0 = immobile or < 50 yards

1 = wheelchair independent, including corners, > 50 yards

2 = walks with help of one person (verbal or physical) > 50 yards

3 = independent (but may use any aid; for example, stick) > 50 yards

STAIRS

0 = unable

1 = needs help (verbal, physical, carrying aid)

2 = independent

Mahoney FI, Barthel DW. Functional evaluation: the Barthel index. Maryland State Medical Journal 1965;14:61-5.

Collin C, Wade DT, Davis S, Horne V.The Barthel ADL index:a reliability study.Int Disabil Studies.1988;10:61-3.

7-item OARS

Instrumental activities of daily living will be described using a short (7-item) questionnaire derived from the Older Americans Resources and Services (OARS) instrument.

Seven items-domains with scoring

Can you use the telephone:
2. Without help, including looking up numbers and dialing.
1. With some help (can answer phone or dial operator in emergency but need a special phone or help in getting the number or dialling).
0. Completely unable to answer the telephone.
- . Not answered.

Can you get to places out of walking distance:
2. Without help (i.e. drive your own car, travel alone on buses or taxis).
1. With some help (need someone to help you or go with you when travelling).
0. Unable to travel unless emergency arrangements are made for a specialized vehicle like an ambulance.
- . Not answered.

Can you go shopping for groceries or clothes (assuming has transportation):
2. Without help (taking care of all shopping needs yourself).
1. With some help (need someone to go with you on shopping trips).
0. Completely unable to do any shopping.
- . Not answered.

Can you prepare your own meals:
2. Without help (plan and cook full meals yourself).
1. With some help (can prepare some things but unable to cook full meals yourself).
0. Completely unable to prepare any meals.
- . Not answered.

Can you do your housework:
2. Without help (can clean floors etc.).
1. With some help (can do light housework but need help with heavy work).
0. Completely unable to do any housework.
- . Not answered.

Can you take your own medicine
2. Without help (in the right doses at the right time).
1. With some help (able to take medicine if someone prepares it for you, reminds you to take it).
0. Completely unable to take medicines.
- . Not answered.

Can you handle your own money
2. Without help (write cheques, pay bills etc).
1. With some help (manage day to day buying but needs help with managing chequebook and paying bills etc.).
1. Completely unable to handle money.
- . Not answered.

Fillenbaum GG, Smyer MA. The development, validity and reliability of the OARS multidimentional functional assessment questionnaire. Journal of Gerontology 1981;36:428-34

Addendum – Revised projected recruitment numbers and implications for conduct and statistical power of the study

In the initial study plans we aimed to recruit 3000 community dwelling subjects aged 65 years or over with sub clinical hypothyroidism. However recruitment rates are much lower than expected (20-25 per month) and it is likely that we will achieve a total of around 540 randomised to the study; with the upscaling of geographical areas for recruitment in all countries we could anticipate a increase of recruitment rate up to a maximum of around 750 patients randomised. While this is substantially fewer patients than the 3000 initially proposed, the study will still contribute substantially to knowledge on treatment of subclinical hypothyroidism in older people.

Revised power calculations:

Given the projections for recruitment revised power calculations have been calculated for total recruitment numbers of 540 and 750, and with mean follow-up of 18 months.

Primary endpoints:

(1) Change in disease specific QOL (measured using symptom and fatigue domains from the Thyroid-specific Quality of Life patient-reported outcome measure (ThyPRO).

Within Thypro we are assessing 3 domains, tiredness, hypothyroid symptoms and hyperthyroid symptoms. Hyperthyroid symptoms are seen as a possible adverse effect. Therefore tiredness and hypothyroid are our efficacy outcomes, and are given equal weights as co-primary outcomes, and split the p-value equally to each (0.05/2=0.025 to each test).

Personal communication from the author of Thypro (Thorquil Watt) indicates that a 9 unit change is a realistic and clinically meaningful effect size.

<u>Power calculations:</u>

Observed SDs for our data at visit 5 (1-year) values adjusted for baseline are 13.3 and 18.3 (on 100-point (%) scales) for hypothyroid and tiredness scales respectively.

We will have 80% power to detect a delta with levothyroxine treatment of 3.5% (3.0%) on the hypothyroid scale with total sample sizes of 540 (750).

We will have 80% power to detect a delta of 4.9% (4.1%) on the tiredness scale with total sample sizes of 540 (750).

Therefore we anticipate we will have good statistical power to detect a clinically meaningful effect of levo-thyroxine on thyroid specific quality of life.

(2) Fatal and non-fatal cardiovascular events (acute myocardial infarction; stroke;

amputations for peripheral vascular disease; revascularisations for atherosclerotic vascular disease, including for acute coronary syndrome and heart failure hospitalisations).

We calculated to detect a Hazard Ratio (HR) of 0.75 for effect of levo-thyroxine we would need 1,034 subjects per group (379 events in total) for 80% power at the 5% level (2-tailed). However it was thought likely that drop-ins/drop outs would reduce the intention-to-treat effect; if the treatment effect was attenuated to a HR of 0.79 then 3,000 subjects (generating 565 events) would give 80% power. This magnitude of benefit is similar to that of aspirin. We therefore intended to recruit 3,000 subjects (1,500 in Levothyroxine arm, 1,500 placebo).

However it is now clear that we will not accrue the required number of vascular events to achieve the above statistical power;

- We will have fewer subjects than initial power calculations, with reduced follow-up and we will accrue a greatly reduced number of vascular events than initially anticipated.
- It is clear that we will be underpowered to detect an effect on incident vascular events.

We are well placed to determine effect of levothyroxine on many of our pre-specified secondary outcomes: indicative power calculations are included below; please note these may be revised if the underlying assumptions do not hold.

Secondary endpoints:

(1) General QOL (measured using EuroQOL) at baseline; 6-8 weeks; 12 months and final follow up.

If we assume SD of 0.32 for change in EuroQOL Score (maximum 1.0), and SD 21.4% for visual analogue scale; based on placebo data from study of frail older people (change over 4 month period); (McMurdo et al JAGS 2009;57:2239);

we would have 80% power to detect delta with levothyroxine of 0.038 (0.033) on EuroQOL Score with total sample sizes of 540 (750);

we would have 80% power to detect delta of 2.57% (2.18%) on visual analogue scale with total sample sizes of 540 (750) .

(2) Handgrip strength (measured using the Jamaar hand dynamometer) at baseline; 12 month and final follow up.

If we assume SD of 3.6 kg for change in handgrip strength; based on placebo data from study of frail older people (change over 4 month period); (McMurdo et al JAGS 2009;57:2239);

we would have 80% power to detect delta of 0.87kg (0.74kg) assuming SD = 3.6 kg with total sample sizes of 540 (750);

we would have 80% power to detect delta of 1.43kg (1.21kg) assuming SD = 5.9 kg (current observed value in TRUST) with total sample sizes of 540 (750).

(3) Executive cognitive function (measured using Letter Digit Coding Test [LDCT) at baseline and final follow-up.

If we assume SD of 3.2 for change in Letter Digit Coding Test; based on placebo data from study of vitamin therapy for frail older people (change over 12 month period); (Stott et al Am J Clin Nutr 2005;82:1320);

we would have 80% power to detect delta of 0.77 (0.66) with total sample sizes of 540 (750).

(4) Total mortality and cardiovascular mortality

We might assume total mortality 5.2% and cardiovascular mortality 2.7% over 18 months of follow-up; based on placebo data from PROSPER; (Shepherd et al Lancet 2002;360:1623).

It is clear that there will be negligible power for this outcome.

(5) Functional ability (basic Activities of Daily Living (ADL) measured using Barthel Index [BI]; extended ADL measured using the older American resources and services [OARS]) at baseline and final follow-up.

If we assume 8% will have a deterioration in Barthel, and 11% a deterioration in extended ADL over 18 month period; based on data from PROSPER (Kamper et al Age and Ageing 2005;34:450);

we would have 17% (23%) power to detect an OR = 0.7 with total sample sizes of 540 (750) for Barthel;

we would have 22% (29%) power to detect an OR = 0.7 with total sample sizes of 540 (750) for extended ADL.

(6) Haemoglobin, measured on a full blood count at baseline and 1 year.

If we assume SD of 0.675 g/dL for change in haemoglobin, based on change over 3 months in levothyroxine treated group in Ravanbod et al (Am J Med 2013;126:420);

we would have 80% power to detect delta of 0.16 g/dL (0.14 g/dL) with total sample sizes of 540 (750).

(7) Blood pressure, measured at screening, 1 year and at final review.

If we assume a SD of 9.7 mmHg for systolic BP, and 7.9 for diastolic blood pressure; based on paired measurements from Vollmer et al J Hum Hypertension 2005;19:77;

we would have 80% power to detect delta of 2.34 mmHg SBP (2.00 mmHg SBP) with total sample sizes of 540 (750);we would have 80% power to detect delta of 1.91 mmHg DBP

(1.62 mmHg DBP) with total sample sizes of 540 (750).

(8) Weight and waist circumference, measured at screening, 1 year and at final review.

If we assume SD of 3.4 kg for change in weight; based on placebo data from study of frail older people (change over 4 month period); (McMurdo et al JAGS 2009;57:2239);

we would have 80% power to detect delta of 0.82 kg (0.70kg) with total sample sizes of 540 (750).

If we assume SD of 5.4cm for change in waist circumference; based on data from cohort study of mid-life women; (Sternfeld et al Am J Epidemiol 2004;160:942);

we would have 80% power to detect delta of 1.30 cm (1.11 cm) with total sample sizes of 540

(750).

Subgroup analyses:

We planned subgroup analyses for gender, age > 85 and < 85 years, known previous thyroid disease and baseline TSH above and below 10 mU/L, as recommended in the Cochrane Systematic Review, and also for known cardiovascular disease at study baseline. We accept even with 3000 recruits that our study will be underpowered for some of the smaller subgroups, such as men, age > 85 years and TSH >10.0, <19.9 mU/L. However we anticipated that we would have sufficient statistical power in the TRUST trial on its own to detect beneficial effects in the larger or dominant subgroups, such as women, age >65, but

<85 years and TSH in the range >4.6, <10.0 mU/L. Given the likely recruitment numbers as listed above it is clear that we will not be adequately powered to detect any beneficial effect in cardiovascular events in any of these subgroups.

STUDY: TRUST EudraCT Number 2011-004554-26

REC reference 11/SS/0071

R&D reference GN11GE272

List of amendments / variations to Protocol **Chief Investigator: Prof. D J Stott** **Page: 1 of 5**

**Amendment number And Sponsor Approval date**	**Substantial / non-substantial**	**Reviewed by REC and / or MHRA**	**Amendment summary**	**Documents Amended (List current versions)**
AMO 1 (22/10/2012)	Substantial	REC and MHRA	Increase of TSH monitoring requested by MHRA.<br>Reduction of starting dose from 50 to 25ug for participants with low weight (<50Kg) or known coronary heart disease.<br>Change of thyroid quality of life questionnaires from the Underactive Thyroid-Dependent Quality of Life and Symptom Rating Questionnaires to the thyroid-related quality of life measure (ThyPRO: hypothyroid symptoms, fatigue and hyperthyroid symptoms	Protocol V3.0<br>Participant Info screening V3.0<br>Participant study Info Sheet V3.0<br>Patient alert card V2

			domains).<br>Tablets rather than capsules to be used.	
AMO 2 (21/02/13)	Non-substantial	REC	Inclusion of Robertson Centre for Biostatistics as data centre to collect participant data.	Protocol V3.1<br>Participant Info screening V3.1,<br>GP letter to patient V 2.0
AMO 3 (30/05/2013)	Non Substantial	REC	Extend recruitment to hospital out-patient clinics	Protocol V3.2<br>Participant Info Screening v3.2
AMO 4 (11/06/2013)	Non Substantial	REC	Extend recruitment to include subjects with recorded biochemistry consistent with subclinical hypothyroid over previous 36 months from original 12	Protocol V3.3
AMO 5 (21/02/2014)	Substantial	REC	3 additional questions on vitality added to the existing 16 ThyPRO questions listed in the protocol.<br>Laboratory flag added to routine thyroid function reporting; alerting GP to patients ≥65yrs with biochemistry consistent with subclinical hypothyroid as potentially eligible for the study.<br>Timing of initial BP measurement changed from baseline to screening visit	Protocol V3.4
AMO 6 (14/03/2014)	Substantial	REC	Addition of Lanarkshire as a Scottish site.	Patient info for screening V3.3<br>Patient info for study V3.2<br>Hospital Dr Letter to Patient V1.0

AMO 7 (11/07/2014)	Substantial	MHRA	Notification of change of manufacturing site for levothyroxine / placebo	
AMO 8 (02/09/2014)	Substantial and non-substantial	REC	Non Substantial:<br>Addition of Tayside, Ayrshire & Arran, Dumfries & Galloway as Scottish sites.<br>Addition of biobank blood sample at one year<br>Substantial:<br>Addition of 'adrenal disorder' to exclusions	Protocol V4.0<br>Patient Info V4.0<br>Consent for Screen V4.0<br>Consent for study V4.0
AMO 9 (12/01/2016)	Substantial and non-substantial (09/03/2016)	REC / MHRA	Substantial<br>Reduction in projected recruitment numbers from 3000 to minimum expected of 540.<br>Change in primary outcome; initially joint co-primary outcome of incident cardiovascular disease and thyroid specific quality of life. Incident cardiovascular disease demoted to secondary outcome as limited statistical power with the reduced recruitment numbers.<br>SmPC revision:<br>Concomitant use of tricyclic antidepressants and levothyroxine may precipitate cardiac arrhythmias.<br>Initiation or discontinuation of anti-convulsant therapy may alter levothyroxine dosage requirements.<br>Effects of levothyroxine may be decreased by concomitant sertraline.	Protocol V5.0<br>Patient Info sheet for study V5.0

			Metabolism of levothyroxine (thyroxine) accelerated by primidone.<br>Imatinib: plasma concentration of levothyroxine (thyroxine) possibly reduced by imatinib.<br>Beta blockers may decrease the peripheral conversion of levothyroxine.<br><br>Non Substantial:<br>1. Reduced period of minimum expected follow up from 24 to 12 months.<br>2. Principle investigators allowed to override dosing algorithm if clinically indicated.	
AMO 10 (10/03/2016)	Substantial	REC	ThyPRO 39 questions added at final visit as secondary outcome.	Protocol V6
AMO 11 (30/05/2016)	Substantial	REC	Final visit – process of unblinding of patient and GP.	Protocol V6.1<br>Letter to Patient re unblinding<br>Letter to GP re unblinding and final TSH

Variation in study protocol in Switzerland	Not applicable – applied before the 1st Swiss participant was randomized	Approved by Swiss ethical board, Swissmedic (the Swiss competent authority for drugs) and by Swiss sponsor	Swiss eligibility criteria amended prior to commencement of the study; patients not required to have fT4 measure at pre-screening, and could be recruited with one fT4 measured within the laboratory reference range (checked in the 6 weeks before randomization). Rationale: GPs in Switzerland routinely review TSH but not always fT4 levels in the follow up of their patients with untreated subclinical hypothyroidism.	Swiss protocol; no other variations
