# Supplementary material for: Effect of Levothyroxine Therapy on the Development of Depressive Symptoms in Older Adults With Subclinical Hypothyroidism: An Ancillary Study of a Randomized Clinical Trial
Source: JAMA Netw Open. 2021 Feb 10;4(2):e2036645. doi: 10.1001/jamanetworkopen.2020.36645 (PMC7876592; doi:10.1001/jamanetworkopen.2020.36645)
Supplement: Supplement 2. — eTable 1. Comparison of Baseline Characteristics Between Participants With Available Follow Up and Participants With Missing Follow Up eTable 2. Secondary Analyses: Incidence of Mild Depression eTable 3. Secondary Analyses: Recovery from Mild Depression eTable 4. Quality of Evidence (GRADE) on Effect on Levothyroxine Therapy in Non-pregnant Adults With Subclinical Hypothyroidism on Depressive Symptoms [file jamanetwopen-e2036645-s002.pdf]

Supplemental Online Content

Wildisen L, Feller M, Del Giovane C, et al. Effect of levothyroxine therapy on the development of depressive symptoms in older adults with subclinical hypothyroidism: an ancillary study of a randomized clinical trial. *JAMA Netw Open*. 2021;4(2):e2036645. doi:10.1001/jamanetworkopen.2020.36645

eTable 1. Comparison of Baseline Characteristics Between Participants With Available Follow Up and Participants With Missing Follow Up

eTable 2. Secondary Analyses: Incidence of Mild Depression

eTable 3. Secondary Analyses: Recovery from Mild Depression

eTable 4. Quality of Evidence (GRADE) on Effect on Levothyroxine Therapy in Non-pregnant Adults With Subclinical Hypothyroidism on Depressive Symptoms

This supplemental material has been provided by the authors to give readers additional information about their work.

© 2021 Wildisen L et al. *JAMA Network Open.*

eTable 1 – Comparison of Baseline Characteristics Between Participants With Available Follow Up and Participants With Missing Follow Up.				
	**Complete Cases**		**Incomplete Cases**	
**Characteristics**	**Levothyroxine Group (N = 211)**	**Placebo Group (N = 216)**	**Levothyroxine (N = 25)**	**Placebo (N = 20)**
Age - yr				
Mean<br>Range	73.99<br>65 - 91	75.04<br>65 - 93	76<br>66 - 88	75.38<br>65 - 87
Female sex – no. (%)	118 (56)	121 (56)	15 (60)	12 (60)
Previous medical conditions and clinical descriptors — no./total no. (%)				
Atrial fibrillation	28 (13)	23 (11)	6 (25)	4 (20)
Hypertension	102 (48)	98 (46)	15 (60)	10 (50)
Diabetes mellitus	34 (16)	26 (12)	4 (16)	2 (10)
Osteoporosis	26 (13)	31 (15)	2 (8)	1 (5)
Current Smoking	18 (9)	19 (9)	2 (8)	3 (15)
Dementia	0 (0)	0 (0)	0 (0)	0 (0)
Excess alcohol consumption*	2 (1)	3 (1)	0 (0)	0 (0)
Antidepressants Medication	16 (8)	10 (5)	5 (20)	3 (15)
Median Mini–Mental State Examination score (SD)	28.52 (1.39)	28.68 (1.42)	28.2 (1.9)	28.5 (2.1)
Weight				
Median weight in kg (range)	77.58 (46 to 150)	76.76 (44 to 121)	74.10 (52 to 112)	73 (48 - 104)
<50 kg — no. (%)	4 (2)	3 (1)	0 (0)	2 (10)
Median BMI (SD)	27.88 (5.54)	27.60 (4.37)	27.32 (4.52)	27 (6)
Thyrotropin — mIU/liter				
Median (SD)	6.57 (2.22)	6.55 (2.04)	6.99 (2.21)	7.33 (3.49)
Range	4.60 to 17.58	4.60 to 17.60	4.90 to 13.38	4.7 to 17.6
Free thyroxine — pmol/liter				
Median (SD)	13.69 (1.97)	13.61 (1.86)	13.84 (2.45)	13.93 (2.65)
Range	10.00 to 20.60	9.00 to 21.90	9.30 to 19.50	10.5 to 19.7
Depressive Symptoms Score, Mean GDS-15 score (SD)	1.26 (1.85, 0 - 9)	0.96 (1.58, 0 - 12)	1.77 (2.52, 0 - 10) †	1.83 (2.36, 0 - 9) †

*more than 35 units per week for female and more than 50 units per week for male [33]

† GDS-15 score at baseline was available for 22 participants from the levothyroxine group and 18 from the placebo group.

Abbreviations: **yr**: year; **N**: Number of participants; **no.**: number; **SD**: standard deviation; **GDS-15**; Geriatric Depression Scale 15-item questionnaire (Range 0-15, higher scores indicate more severe depressive symptoms, minimal clinical important difference: 2 points).

eTable 2 - Secondary Analyses: Incidence of Mild Depression						
	**No. of eligible participants ***, at baseline		**No. of participants with mild depression†**, at 12 months			
	**Levothyroxine**	**Placebo**	**Levothyroxine**	**Placebo**	**Odds Ratio (95% CI) ‡**	**P Value**
CH & NL	188	206	10	12	0.87 (0.36 to 2.13)	0.76
CH, NL & IR	235	246	12	12	1.06 (0.45 to 2.49)	0.89

* Participants with values bellow cut-off (GDS-15 =< 3 resp. CESD-20 =< 20)

† Participants with values above cut-off (GDS-15 > 3 resp. CESD-20 > 20)

‡Odds ratio < 1 indicate benefit for levothyroxine. Adjusted for depressive symptoms score at baseline, sex, age, levothyroxine dose at baseline, country

Abbreviations: **CH,** Switzerland; **NL,** the Netherlands; **IR**; Ireland; **CI**, Confidence Interval; **No.** Number.

© 2021 Wildisen L et al. *JAMA Network Open.*

eTable 3 - Secondary Analyses: Recovery from Mild Depression						
	**No. of eligible participants ***, at baseline		**No. of participants without mild depression**†, at 12 months			
	**Levothyroxine**	**Placebo**	**Levothyroxine**	**Placebo**	**Odds Ratio (95% CI)** ‡	**P Value**
CH & NL	23	10	9	6	0.20 (0.02 to 1.93)	0.16
CH, NL & IR	23	10	9	6	0.20 (0.02 to 1.93)	0.16

* Participants with values above cut-off (GDS-15 > 3 resp. CESD-20 > 20)

† Participants with values below cut-off (GDS-15 <= 3 resp. CESD-20 <= 20)

‡Odds ratio > 1 indicate benefit for levothyroxine, adjusted for depressive symptoms score at baseline, sex, age, levothyroxine dose at baseline, country

Abbreviations: **CH,** Switzerland; **NL,** the Netherlands; **IR**; Ireland; **CI**, Confidence Interval; **No.** Number.

eTable 4 – Quality of Evidence (GRADE) on Effect on Levothyroxine Therapy in Non-Pregnant Adults With Subclinical Hypothyroidism on Depressive Symptoms									
Certainty assessment						No. of patients		Effect	Certainty
No. of studies	Study design	Risk of bias*	Inconsistency	Indirectness	Imprecision	Levothyroxine	Placebo	Absolute (95% CI)	
5	randomised trials	not serious	not serious	not serious	not serious	345	360	SMD **0.09 SD higher**† (0.05 lower to 0.22 higher)	⊕⊕⊕⊕ HIGH ‡

*Risk of Bias assessment of the TRUST trial [7]: Random sequence generation = Low risk, allocation concealment = Low risk, Blinding of participants and personnel= Low risk, Blinding of outcome assessment = Low risk, Incomplete outcome data = Low risk, Selective outcome reporting = Low risk.

†higher depressive symptoms scores in participants with levothyroxine, positive values indicate benefit of placebo.

‡ Further research is very unlikely to change our confidence in the estimate of effect.

Abbreviations: **CI**: Confidence Interval, **No.**: Number, **SMD**: Standardized mean difference

© 2021 Wildisen L et al. *JAMA Network Open.*
