# Supplementary material for: Effect of Levothyroxine Therapy on the Development of Depressive Symptoms in Older Adults With Subclinical Hypothyroidism: An Ancillary Study of a Randomized Clinical Trial
Source: JAMA Netw Open. 2021 Feb 10;4(2):e2036645. doi: 10.1001/jamanetworkopen.2020.36645 (PMC7876592; doi:10.1001/jamanetworkopen.2020.36645)
Supplement: Supplement 3. — Data Sharing Statement [file jamanetwopen-e2036645-s003.pdf]

Data Sharing Statement

Wildisen. Effect of Levothyroxine Therapy on the Development of Depressive Symptoms in Older Adults With Subclinical Hypothyroidism. *JAMA Netw Open*. Published February 10, 2021. doi:10.1001/jamanetworkopen.2020.36645

Data
Data available: No

Additional Information
Explanation for why data not available: Request for data must be sent to the corresponding author (nicolas.rodondi@biham.unibe.ch)
